# Chronosequence of invasion reveals minimal losses of population genomic diversity, niche expansion, and trait divergence in the polyploid, leafy spurge

**DOI:** 10.1111/eva.13593

**Published:** 2023-10-04

**Authors:** Thomas A. Lake, Ryan D. Briscoe Runquist, Lex E. Flagel, David A. Moeller

**Affiliations:** ^1^ Department of Plant and Microbial Biology University of Minnesota St. Paul Minnesota USA; ^2^ Gencove Long Island City New York USA

**Keywords:** adaptation, colonization bottlenecks, plant invasion, population genetic structure, range shift, rapid evolution

## Abstract

Rapid evolution may play an important role in the range expansion of invasive species and modify forecasts of invasion, which are the backbone of land management strategies. However, losses of genetic variation associated with colonization bottlenecks may constrain trait and niche divergence at leading range edges, thereby impacting management decisions that anticipate future range expansion. The spatial and temporal scales over which adaptation contributes to invasion dynamics remain unresolved. We leveraged detailed records of the ~130‐year invasion history of the invasive polyploid plant, leafy spurge (*Euphorbia virgata*), across ~500 km in Minnesota, U.S.A. We examined the consequences of range expansion for population genomic diversity, niche breadth, and the evolution of germination behavior. Using genotyping‐by‐sequencing, we found some population structure in the range core, where introduction occurred, but panmixia among all other populations. Range expansion was accompanied by only modest losses in sequence diversity, with small, isolated populations at the leading edge harboring similar levels of diversity to those in the range core. The climatic niche expanded during most of the range expansion, and the niche of the range core was largely non‐overlapping with the invasion front. Ecological niche models indicated that mean temperature of the warmest quarter was the strongest determinant of habitat suitability and that populations at the leading edge had the lowest habitat suitability. Guided by these findings, we tested for rapid evolution in germination behavior over the time course of range expansion using a common garden experiment and temperature manipulations. Germination behavior diverged from the early to late phases of the invasion, with populations from later phases having higher dormancy at lower temperatures. Our results suggest that trait evolution may have contributed to niche expansion during invasion and that distribution models, which inform future management planning, may underestimate invasion potential without accounting for evolution.

## 1 INTRODUCTION

Invasive species experience considerable changes in genetic variation during the process of introduction and subsequent invasion (Lee, 2002; Suarez & Tsutsui, 2008). In particular, it has been well documented that founder effects and genetic drift can cause substantial losses of genetic diversity during the colonization process (Dlugosch & Parker, 2008; Uller & Leimu, 2011). Following initial establishment, further losses of variation may occur during range expansion. However, the magnitude of changes in genetic variation depends upon the number of introductions and the severity of population bottlenecks (Nei et al., 1975; Uller & Leimu, 2011; Welles & Dlugosch, 2019). Such changes in genetic variation early in the invasion process may influence the capacity for adaptation, forecasts of range expansion, and subsequent management decisions.

Following colonization, some non‐native species exhibit rapid population growth and dispersal into new environments (Sakai et al., 2001). The process of invasion is often highly variable, involving repeated founder events and density‐dependent population growth, especially Allee effects (Melbourne & Hastings, 2009; Sullivan et al., 2017). In addition to affecting the speed of invasion, these population fluctuations can influence levels of genetic diversity and structure across an invaded range (Austerlitz et al., 1997; Excoffier, 2004). For example, range expansion is expected to cause a reduction in allelic richness and heterozygosity with increasing distance from the origin of expansion (Peter & Slatkin, 2013; Slatkin & Excoffier, 2012). The prevalence of drift during invasion may also cause populations to depart from migration‐drift equilibrium, resulting in a lack of isolation by distance (IBD) (Hutchison & Templeton, 1999; Slatkin, 1987, 1993; Wright, 1943). Last, mutations arising at the range edge may rise in frequency due to genetic drift, “surf” along the expanding front, and travel long distances (Excoffier & Ray, 2008; Klopfstein et al., 2006). Models of allele surfing indicate that rapid range expansion can produce clinal variation in allele frequencies and increase the frequency of loci with private alleles (Excoffier & Ray, 2008; Goodsman et al., 2014; Klopfstein et al., 2006). Such clines can emerge for any type of mutation (beneficial, neutral, deleterious), and therefore could reflect drift and/or selection (Koski et al., 2019; Lehe et al., 2012; Peischl et al., 2013). Overall, the prevalence of drift during range expansion has the potential to influence the capacity for adaptation as organisms encounter novel environments, particularly when functionally important allelic variation is lost.

An increasing body of evidence suggests that rapid phenotypic evolution can be important to the process of range expansion (Colautti & Barrett, 2013; Hodgins et al., 2018; Ma et al., 2020; Selechnik et al., 2019). Forecasts of range expansion in invasive species can underpredict the potential extent of invasion if they assume a species does not evolve or adapt over short time scales (Chardon et al., 2020; Collart et al., 2020). A recent meta‐analysis indicated that the signature of local adaptation in invasive species was at least as strong as in native species, even when accounting for variation in life history (Oduor et al., 2016). Invasive species frequently expand across strong environmental gradients and into novel niche spaces (Atwater et al., 2018; Bates & Bertelsmeier, 2021). Such niche expansion may require adaptive evolution at the invasion front (Chown et al., 2015; Hodgins et al., 2018; Moran et al., 2017). For example, purple loosestrife rapidly diverged in flowering time and plant size during invasion across latitudinal gradients in growing season length (Colautti & Barrett, 2013). Despite evidence of rapid evolution in some systems, range expansion may not involve any changes in the organism's niche if the only limits to spatial expansion are dispersal and time. As such, the apparent expansion of the climate niche with invasion may not actually involve the evolution of ecologically important traits. While tests of local adaptation within an invaded range remain few, there is growing appreciation that rapid evolution is likely to shape the trajectory of range expansion in non‐native species (Hodgins et al., 2018; Woods & Sultan, 2022).

Phenotypic evolution during range expansion may be caused by spatially variable selection and/or neutral processes (e.g., spatial sorting) (Colautti & Lau, 2015; Hodgins et al., 2018; Keller & Taylor, 2008). Adaptive evolution, in particular, may be paramount to the invasion process if selection in response to novel environments results in trait changes that enhance a species' capacity to establish in new habitats (Hodgins et al., 2018; Prentis et al., 2008; Williams et al., 2016; Woods & Sultan, 2022). While reciprocal transplant experiments are the gold standard for testing for adaptation, the translocation of invasive species for these experiments is subject to ethical concerns and legal restrictions in many areas. Alternatively, researchers have started to use ecological niche models (ENMs) to identify important environmental gradients that span from optimal to marginal habitat, such as from a range core to an edge. Predictions are then made about traits that may promote adaptation to the novel environments found in marginal habitats (Capblancq et al., 2020; Dixon & Busch, 2017; Morente‐López et al., 2022; Searcy & Shaffer, 2016). Finally, common garden experiments can determine whether the putative traits under selection have differentiated across the key environmental gradients identified by ENMs. Taken together, this series of approaches can provide insight into the role of adaptation in the process of niche expansion at leading range edges.

Among invasive plant species, polyploidy is prevalent (Pandit et al., 2011) and can influence the process of range expansion (Van de Peer et al., 2021). The frequency of polyploid species increases with higher latitudes, lower temperatures, and seasonally drier environments (Brochmann et al., 2004; Rice et al., 2019). Direct effects of polyploidy on physiological, morphological, and phenological traits may facilitate niche shifts (Blaine Marchant et al., 2016; Brittingham et al., 2018; Glennon et al., 2014; Wang et al., 2022) and preadapt polyploids to new environments (Lachmuth et al., 2010; Treier et al., 2009). Polyploidy may also influence the capacity for adaptation during range expansion as new environments are encountered (Baniaga et al., 2020; te Beest et al., 2012). Although genetic drift during range expansion can cause losses of genetic diversity, drift may have less severe effects (e.g., inbreeding depression) in polyploids relative to diploids when there is polysomic inheritance (Moody et al., 1993; Soltis & Soltis, 2000). Despite numerous polyploid invaders, they have been the subject of few studies because of substantial challenges with the application of evolutionary genetic analyses that were developed for diploids (Rutland et al., 2021).

In this study, we used a well‐documented chronosequence of invasion to examine the consequences of range expansion for population genomic diversity, climatic niche breadth, and the evolution of germination behavior in the polyploid leafy spurge (*Euphorbia virgata*). We focused on one area of introduction to southwestern Minnesota, U.S.A., and subsequent range expansion to the north and east. We were interested in examining the severity of losses of genetic diversity following introduction and their potential consequences for invasion, particularly since existing species distribution models predict a low probability of range expansion at the current leading range edge (Lake et al., 2020). Introduction to this region occurred in the 1890s in southwestern Minnesota, with subsequent range expansion to northeastern Minnesota (ca., 500 km), where populations are currently rare, isolated, and small. Based on a detailed historical occurrence dataset, we defined a range core, an area of early expansion, an area of late expansion, and an invasion front.

First, we sampled populations along the gradient from the core to the invasion front using two sampling schemes to quantify population genomic diversity and structure (using reduced representation sequencing). Samples of multiple individuals from 14 populations (population samples) allowed us to quantify changes in sequence diversity among populations over the course of range expansion. Samples of single individuals from 157 populations (landscape samples) allowed us to test for fine‐scale population structure (e.g., IBD) over the time series of range expansion. Second, we tested whether range expansion, involved niche expansion – i.e., occurred in novel climatic environments. Third, we developed an ENM to test whether habitat suitability declines from range core to invasion front and to determine which environmental gradients are most strongly associated with high versus low habitat suitability. Warm‐ season temperatures had the greatest positive contribution to habitat suitability. Because temperature is known to modulate germination behavior in leafy spurge and because establishment in new habitats is dependent upon successful germination timing, we focused on this trait for common garden experiments. Past work has also suggested that shifts in seed dormancy might facilitate invasion at leading range edges (Mathias & Kisdi, 2002; Travis et al., 2021). We examined the responses of seeds from early versus late invasion to five temperature regimes in a common garden experiment. We specifically tested whether there was an interaction between geographic region (early vs. late invasion stages) and temperature regime, which would indicate divergence in germination behavior over the course of invasion.

## 2 METHODS

### 2.1 Invasion and natural history of leafy spurge

#### 2.1.1 Natural history

Leafy spurge has invaded approximately two million hectares across the northern tier of the United States and southern Canada (Duncan et al., 2004). While it is most commonly found in dry, open sites with well‐drained soils (e.g., prairies), it can occasionally occur in seasonally wet meadows and riparian areas (Selleck et al., 1962). Leafy spurge impacts rangelands and natural habitats by competitively displacing native species (Hein & Miller, 1992). When damaged, plants exude a toxic latex that deters grazing (Lym, 1998; Lym & Kirby, 1987).

Leafy spurge spreads locally via rhizomes and ballistic seed dispersal (Morrow, 1979). Longer distance dispersal has been proposed to occur via animals or agricultural machinery (Lacey et al., 1992; Pemberton, 1988). Seeds germinate in the spring or may remain dormant in soil for at least two years (Hanson & Rudd, 1933; Selleck et al., 1962). Flowers are insect‐pollinated and the mating system is primarily outcrossing (Selleck et al., 1962).

Leafy spurge is an auto‐allohexaploid that likely originated from hybridization between closely related *Euphorbia* species, although the progenitor species are not yet known (Riina et al., 2013; Schulz‐Schaeffer & Gerhardt, 1989). It has been the subject of several genomic investigations but lacks a full genome assembly and annotation (Chao et al., 2005; Horvath et al., 2015, 2018; West et al., 2023).

#### 2.1.2 Invasion history

One introduction of leafy spurge occurred in southwestern Minnesota ca., 1890, purportedly via contaminated grains imported from southern Russia (Batho, 1932; Dunn, 1985; Hanson & Rudd, 1933). Following introduction, the range expanded to eastern South Dakota by ca., 1902 (Bakke, 1936), eastern North Dakota by ca. 1909 (Hanson & Rudd, 1933), and southern Manitoba and Saskatchewan by the 1920s (Batho, 1932; Selleck et al., 1962). Hanson and Rudd (1933) documented in detail the distribution of leafy spurge across Minnesota and neighboring regions, providing a baseline for understanding the timeline of subsequent range expansion (Figure S1). By the late 1970s, leafy spurge had become common throughout the grasslands of the north‐central plains (Dunn, 1979, 1985). The invasion of the boreal forest region of northeastern Minnesota began in the 1940s and 1950s with isolated occurrences (Lakela, 1965) and populations were not common until the 1990s. This invasion front has persisted, with limited expansion furthernortheast. Leafy spurge is uncommon to the west of this invasion front (between Minnesota and western North Dakota), making it unlikely that genotypes from a separate introduction have contributed to genetic variation in this region.

#### 2.1.3 Delineating the timeline of range expansion

**FIGURE 1:**
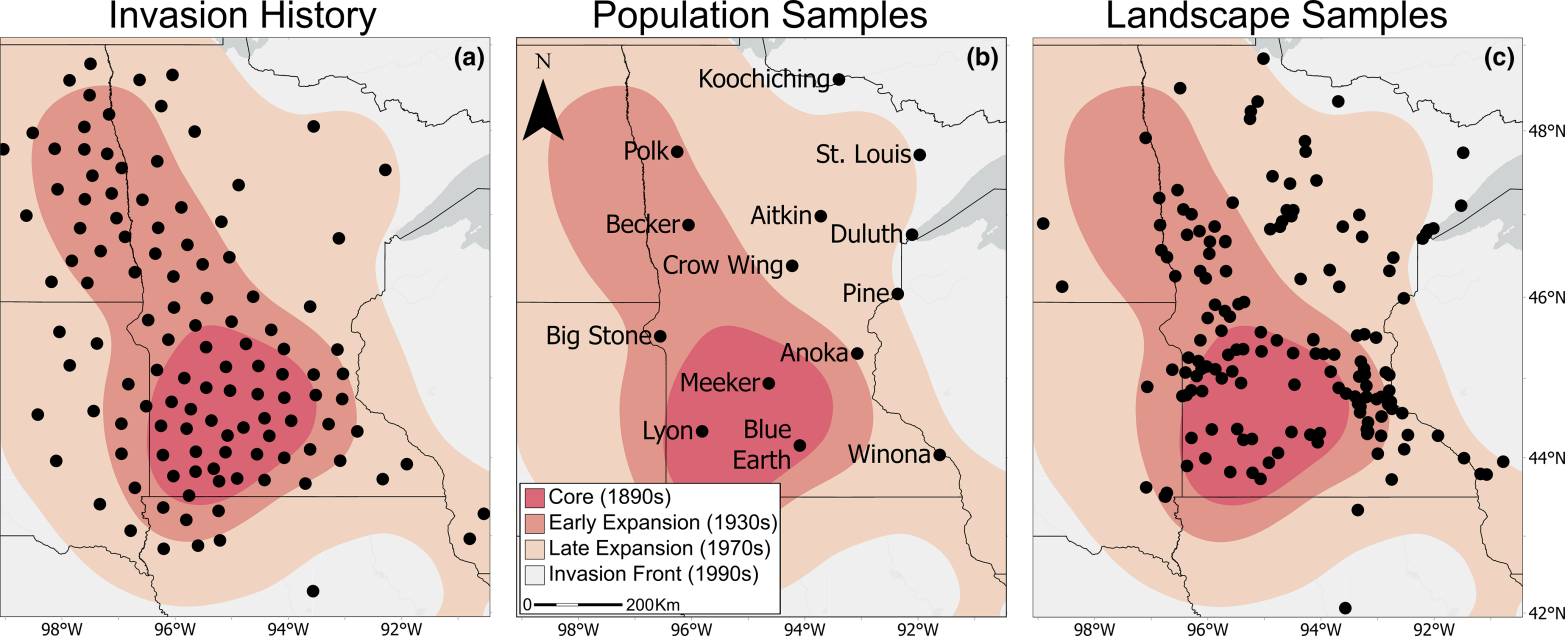
a) Invasion history reproduced from Hanson and Rudd (1933). The map was used to delineate four phases of invasion: core, early expansion, late expansion, and invasion front. Tissue collection sites for b) 14 population samples and c) 157 landscape samples of leafy spurge.

We digitized the earliest known point record map (Hanson & Rudd, 1933) using ArcGIS Pro (ESRI, 2022). We then applied empirical Bayesian kriging to produce a continuous density surface that represented the density of populations in the north‐central plains. From this density surface, we applied an equal‐interval threshold to demarcate a range core, area of early expansion, area of late expansion, and invasion front that corresponded to four density categories across Minnesota and surrounding states (Figure 1a). We verified these demarcations with published accounts of the invasion history (described above).

### 2.2 Population genomic diversity and structure

#### 2.2.1 Sampling and sequencing

In 2019, we collected leaf tissue from six individuals in each of 14 populations distributed evenly across Minnesota (*hereafter*: population samples; Figure 1b). In addition, we collected tissue from one individual in each of 157 populations distributed relatively evenly across Minnesota, eastern South Dakota, eastern North Dakota, and western Wisconsin (*hereafter*: landscape samples; Figure 1c). We sampled tissue from individuals that were at least five meters apart to minimize collecting from the same genet and placed tissues immediately in silica for preservation until DNA extraction (Table S1).

We extracted DNA using QIAGEN DNeasy Plant Mini Kits (QIAGEN Inc.). Dual‐indexed GBS (genotyping‐by‐sequencing) libraries were created using the BamHI + NsiI enzyme combinations. All libraries were pooled and sequenced on an Illumina NovaSeq System (Illumina Inc.) with 1 × 100‐bp sequencing. Once sequenced, the reads were demultiplexed and balanced with a mean quality score ≥Q30 for all libraries. We filtered low‐quality bases using Trimmomatic (Bolger et al., 2014) and used Stacks v.2.5.9 (Rochette et al., 2019) to build loci de novo (i.e., without aligning reads to a reference genome). Overall, we obtained 510 million reads across the 241 samples (599,386–3,376,078 of raw reads per individual). The mean read depth per locus ranged from 14× to 26× (Supplementary Methods S1).

We called SNPs using polyRAD v.2.0.0 (Clark et al., 2019), a Bayesian algorithm designed for polyploid GBS data. PolyRAD estimates the genotype probabilities for each individual from read depth distributions with ploidy level as a prior. First, we filtered our dataset using the H_ind_/H_e_ statistic to cull likely paralogous loci (Clark et al., 2022). Next, we estimated posterior probabilities for each genotype using the ‘IterateHWE’ function with Hardy–Weinberg equilibrium as the prior (Clark et al., 2022; Gerard & Ferrão, 2020). For each individual at each locus, we exported the most probable genotype for subsequent analyses (Supplementary Methods S1).

We implemented a second filtering step to account for potential biases caused by homoeologous loci present in our dataset. Because leafy spurge is an auto‐allohexaploid (Schulz‐Schaeffer & Gerhardt, 1989), we expect homoeologous loci to have a 2:1 allelic ratio (e.g., AAAABB genotype; Horvath et al., 2018). Indeed, we identified a peak in the minor allele frequency spectrum around 0.33 (Figure S2). We removed loci with a minor allele frequency above 0.26 from our dataset because they are likely to have an excess of homoeologous genotypes (Figure S2). While essential, this second filtering step limits our capacity to understand absolute levels of sequence diversity. However, our primary goal was to examine changes in sequence diversity over the course of the invasion rather than absolute quantities. After filtering, 3176 loci remained for downstream analyses.

#### 2.2.2 Population structure

We performed an analysis of molecular variance (AMOVA) to quantify the proportion of genetic variation partitioned among populations, among individuals within populations, and within individuals (Excoffier et al., 1992; Meirmans, 2012, 2020). We estimated genetic variance components using the rho statistic (Ronfort et al., 1998) and determined significance using permutation tests (*n* = 999) using the R package poppr v.2.8.6 (Kamvar et al., 2015). Further, we checked for clonality among samples using the ‘clonecorrect’ function in (Kamvar et al., 2015).

We assessed population structure using the Bayesian clustering algorithm STRUCTURE v.2.3.4 (Pritchard et al., 2000). We ran the analysis 10 times for each of K = 1–10 with 500,000 Markov Chain Monte–Carlo iterations with a 50,000‐run burn‐in period, specifying an admixture model with the assumption of uncorrelated allele frequencies. We used ‘structure_threader’ in Python 3 (Pina‐Martins et al., 2017) to parallelize runs across clusters (K = 1–10). We determined the most plausible number of clusters using the ΔK method (Evanno et al., 2005) and STRUCTURE HARVESTER web v.0.6.94 (Earl & vonHoldt, 2012).

We performed principal component analyses (PCA) to examine population structure using GENODIVE v.3.0.4 (Meirmans, 2020). We performed PCA separately for the population samples and the landscape samples.

We tested for IBD (Wright, 1943) in the population samples by estimating genetic differentiation as G_ST_ (Dufresne et al., 2014) using GENODIVE v.3.0.4 (Meirmans, 2020). We also tested for IBD in the landscape samples by calculating Nei's genetic distance (Nei, 1972) (Meirmans, 2020). We subset the landscape samples according to successive stages of range expansion and tested for IBD within each subset (i.e., within the range core, then successively including areas of early expansion, late expansion, and invasion front). We tested for a relationship between the pairwise genetic and geographic distance (kilometers) matrices using Mantel tests with 9999 permutations in the R package ‘adegenet’ v.2.1.8 (Jombart, 2008).

#### 2.2.3 Tests for changes in population genetic diversity during range expansion

For each of the 14 population samples, we estimated the inbreeding coefficient (G_IS_) and observed heterozygosity (H_o_; gametic heterozygosity; Moody et al., 1993), which corrects for potential overestimates of heterozygosity in polyploids by calculating the fraction of heterozygotic gametes for each genotype using GENODIVE v.3.0.4 (Meirmans, 2020). We also calculated the number of private alleles (P) using poppr v.2.8.6 (Kamvar et al., 2015). We calculated Tajima's *D* (Tajima, 1989) using DNASp v.6.0 (Rozas et al., 2017) to gauge if populations have an excess or deficit of rare alleles, which can be indicative of population expansion following a bottleneck (negative *D*) or sudden population contraction (positive *D*), respectively. Because we filtered loci with higher minor allele frequencies, Tajima's *D* should be biased toward lower values. Finally, we tested for evidence of genetic bottlenecks based on linkage disequilibrium using NeEstimator v.2.0.1 (Do et al., 2014).

We tested for changes in population genetic parameters (H_o_, G_IS_, P, and Tajima's *D*) as populations dispersed beyond the range core (Table 1). For each statistic separately, we used a multiple linear regression that included latitude, longitude, and their interaction as independent variables with the R package ‘car’ v.3.1‐2 (Fox & Weisberg, 2019). As all late expansion and invasion front populations are located either north or east of the range core, latitude and longitude describe the northern and eastern invasion spreads, respectively. In the model of private alleles, we identified the ‘Winona' population as an outlier using diagnostic plots of residuals (Figure S3), so we removed it from the analysis. We were unable to conduct such a geographic test of N_e_ from the NeEstimator analyses because all but three populations had an N_e_ of infinity (Table S2).

**TABLE 1:** Locality information and genetic diversity metrics (observed heterozygosity, Ho; inbreeding coefficient, G_is_; number of private alleles, and Tajima's *D*) for 14 leafy spurge population samples from four phases of range expansion (see Figure 1).

Range Position	Population Genetic Samples	Latitude	Longitude	Observed Heterozygosity (Ho)	Inbreeding Coefficient (G_is_)	Private Alleles	Tajima's *D*
Core	Blue Earth	44.16	−94.09	0.068	0.123	27	−0.037
	Lyon	44.33	−95.82	0.067	−0.017	9	−0.020
	Meeker	44.94	−94.64	0.07	−0.017	4	0.205
Early Expansion	Becker	46.88	−96.05	0.068	0.116	10	−0.052
	Big Stone	45.52	−96.55	0.067	0.127	9	−0.028
	Polk	47.75	−96.25	0.07	0.097	11	−0.058
Late Expansion	Aitkin	46.98	−93.72	0.068	0.138	27	0.122
	Anoka	45.29	−93.13	0.071	−0.026	7	0.101
	Crow Wing	46.38	−94.22	0.065	0.081	8	0.001
	Winona	44.04	−91.62	0.071	0.156	42	−0.167
Invasion Front	Duluth	46.76	−92.11	0.066	0.12	23	−0.034
	Koochiching	48.60	−93.40	0.066	0.089	10	−0.008
	Pine	46.04	−92.36	0.069	0.138	21	0.000
	St. Louis	47.72	−91.97	0.065	0.046	6	0.068

### 2.3 Niche breadth and habitat suitability

#### 2.3.1 Environmental data

We downloaded 19 bioclimatic variables at a 30 arcsecond resolution (~1 km) from Worldclim (http://worldclim.org/version2). We used a principal component analysis based on the 157 landscape samples to examine patterns of correlation among bioclimatic variables. The biplot of the first two principal components (74.7% variance explained) showed that there were three sets of correlated variables: one set includes mostly precipitation variables, a second set includes mostly temperature variables, and a third set includes variables that describe seasonality and temperature ranges (Figure S4). Because the selection of one variable from a set of correlated ones is challenging (e.g., choosing one precipitation variable from a set of correlated precipitation variables), studies have advocated for choosing variables that are biologically most relevant to the organism and largely uncorrelated (Chapman et al., 2017; Petitpierre et al., 2017).

We selected the minimum temperature of the coldest month (Bio 6), the mean temperature of the warmest quarter (Bio 10), and the precipitation of the warmest quarter (Bio 16). These three variables provide biologically meaningful axes of climate variation that are relevant to key life‐history stages for an herbaceous perennial (Chapman et al., 2017; Petitpierre et al., 2017). Specifically, minimum cold temperatures are important for overwintering and cold tolerance (Chapman et al., 2017), whereas the temperature of the warmest quarter likely influences seed germination, plant growth, and phenological transitions (Wolkovich et al., 2013). Precipitation in the warmest quarter likely also affects plant growth and reproduction, with lower precipitation associated with reduced growth and increased drought stress in northern temperate ecosystems (Gorton et al., 2019; Petitpierre et al., 2017).

#### 2.3.2 Tests for niche differentiation during range expansion

We tested for climatic niche differentiation between the range core, early expansion, late expansion, and invasion front using the ‘ecospat’ R package v.3.5.1 (Di Cola et al., 2017). We sampled the total extent of the background environmental space with a principal component analysis using 1500 random points drawn from a bounding box centered on Minnesota (Latitude: min: 43° N, max: 50° N; Longitude: min: −98° W, max: −89° W). We then used the landscape sample localities to calculate the niche boundaries and density of occurrence for each portion of the range within the environmental PCA space.

For all pairwise comparisons of the range core, early expansion, late expansion, and invasion front, we quantified four measures of niche differentiation. First, we used Schoener's *D* to quantify the similarity in niche by incorporating both niche breadth and density (Warren et al., 2008). Values of *D* can vary from 0 (no overlap) to 1 (complete overlap). For each pair of regions, we then calculated what proportion of the combined niche space represented niche stability, expansion, and unfilling. In this framework, if ‘A' represents the older portion of the range (range core) and ‘B' represents the more recent portion of the range (invasion front), niche stability is the proportion of niche B that overlaps A, niche expansion is the proportion of niche B that does not overlap A, and niche unfilling is the proportion niche A that does not overlap B.

We used permutation tests to determine if the values of Schoener's *D*, expansion, stability, and unfilling were equivalent between the range core, early expansion, late expansion, and invasion front. In the niche equivalency tests, the data were pooled and then randomly assigned to one group of the pairwise range comparisons for 999 permutations. For each permutation, we computed all four statistics. We rejected the null hypothesis of niche equivalency if the observed Schoener's *D* value was less than 95% of the permuted *D* values. Similarly, we rejected niche equivalency based on the combined niche space if observed stability was less than 95% of permuted values and observed niche expansion or unfilling was greater than 95% of permuted values.

#### 2.3.3 Ecological niche model

We developed an ENM using MaxEnt v.3.4.3 (Merow et al., 2013; Phillips et al., 2017) with the ‘dismo’ package v.1.3‐14 in R (Hijmans et al., 2022). Our goal was to identify environmental gradients that could potentially drive phenotypic evolution during range expansion (Araújo et al., 2019; Elith & Leathwick, 2009; Morente‐López et al., 2022). We built ENMs with the same bioclimatic variables and in the same bounding box as the analyses of niche differentiation. We found that the temperature of the warmest quarter (Bio10) had the strongest influence on the probability of occurrence. To examine whether this result was robust, we built three other ENMs using alternative sets of environmental variables (Table S3). In every case, the temperature of the warmest quarter was by far the strongest predictor (Table S3).

We built all ENMs using occurrence records from our tissue collection sites and 10,000 background points. We excluded threshold and hinge features during the model building process as the preliminary models that included these features tended to be overspecified. We used five‐fold cross‐validation to assess model performance; data were randomly partitioned into five equal groupings, and 80% of the data were used for training and 20% for evaluation. Model predictions are the mean of the five cross‐validated models.

We evaluated models using AUC and sensitivity, which were calculated using the withheld dataset. AUC characterizes model discrimination ability and ranges between 0 and 1, with higher values indicating greater model performance and a value of 0.5 indicating that model discrimination is no better than random (Phillips & Dudík, 2008). Sensitivity quantifies the proportion of correctly identified positives. We calculated sensitivity where the sum of the true positive rate and true negative rate was maximized (threshold = 0.53). We then used the variables permutation importance and percent contribution to identify which environmental variable had the greatest contribution to habitat suitability. We also visualized response curves for each environmental variable to ensure that models were not overspecified. Hereafter, “habitat suitability” refers to the probability of occurrence from our ENMs based upon climatic factors.

To test for divergence in germination behavior during range expansion, we focused on the mean temperature of the warmest quarter since it most strongly affected predicted habitat suitability in all ENMs. Populations from early in the invasion had high predicted habitat suitability and higher warm season temperatures (all above 20°C) compared to those from later in the invasion, which had lower predicted habitat suitability and lower warm season temperatures (all below 20°C; Figure 6a,b). Guided by these results, we divided the 14 populations for which we had seed collections into two groups: early (*n* = 8) versus late (*n* = 6) in invasion, and exposed them to five temperature regimes (see below).

### 2.4 Tests for differentiation in germination behavior during range expansion

We collected seeds in 2019 from 14 populations (8–24 maternal families/pop; Table S4). Seeds were collected from individuals that were at least 5 meters apart. We stored and after‐ripened seeds in an indoor environment for 2 years prior to the germination experiment (Wicks & Derscheid, 1958). Seeds were pooled within populations prior to applying treatments. We were unable to collect seeds from every population used in the population genetic survey because some had already dispersed seeds prior to our collection effort.

We examined the effects of temperature regime and source geographic region (early vs. late invasion) on germination. Five temperature regimes were designed to mimic the full range of variation in daytime and nighttime temperatures during the spring and summer in this region (14 h day/10 h night periods: 15/5°C, 20/10°C, 25/15°C, 30/20°C, and 35/25°C). The experiment was conducted in five successive rounds in two growth chambers (Conviron Inc.). We conducted each temperature regime twice – i.e., once in each growth chamber – to control for growth chamber effects.

For each round, we placed ten seeds per population in 60 × 15 mm polystyrene Petri dishes containing 2 mL sterile distilled water, lined with one Whatman #1 filter paper, and sealed with parafilm. Each population was replicated three times per chamber for a total of 42 dishes per treatment per chamber or 84 dishes per round. Dishes were wrapped in aluminum foil to block light, which can inhibit germination (Selleck et al., 1962). Every 24 h, we recorded the number of germinated seeds (emergence of radicles) per dish. After each treatment period ended, we tested whether ungerminated seeds were viable by soaking bisected seeds in a 1% tetrazolium solution for 24 h (Verma & Majee, 2013). Red staining of tissues indicates that seeds are viable. For analyses of germination, we included the number of germinated seeds out of the total number of germinated plus viable (but ungerminated) seeds.

We tested for the effects of temperature regime (categorical), source geographic region, and their interaction on germination probability using a mixed‐effects model with a binomial family. Experimental round and population were included as random effects. We evaluated significance with Type III tests. All models were run using the ‘mixed’ function in the ‘afex’ package v.1.3‐0 (Singmann et al., 2016) in R v.4.0.2 (R Development Core Team, 2015). We used linear contrasts to test for differences in germination between geographic regions for each temperature regime category individually.

## 3 RESULTS

### 3.1 Population genomic consequences of range expansion

**FIGURE 2:**
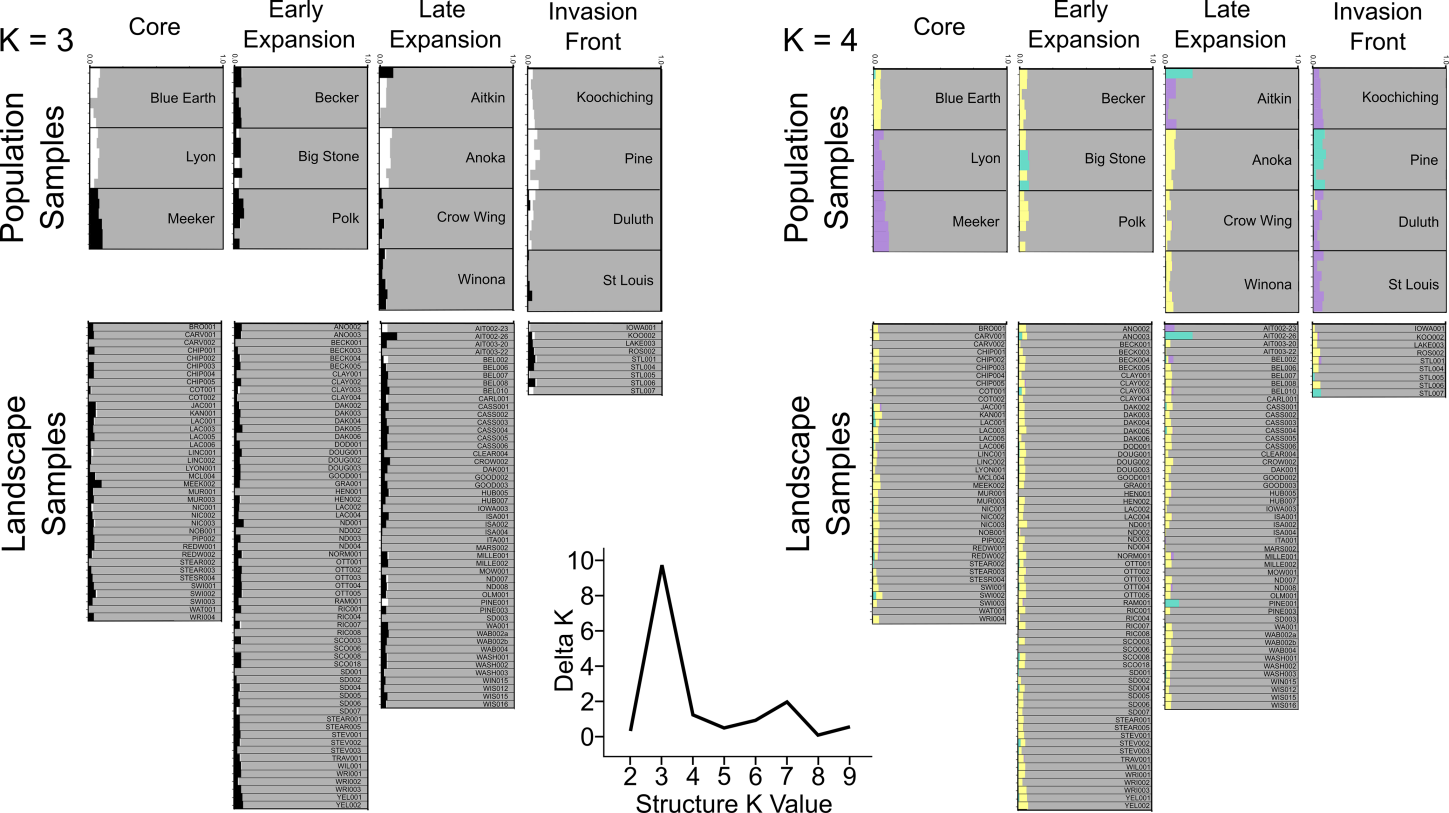
Cluster assignment probability from STRUCTURE analyses (K = 3 and K = 4) for population and landscape samples. Each bar represents one individual, and populations are separated by black lines. A graph of the delta K values supports the optimal number of clusters at K = 3.

Analysis of molecular variance (AMOVA) revealed significant partitioning of genetic variance among populations (13.7%; *p* < 0.001), among individuals within populations (8.4%; *p* < 0.001), and within individuals (77.8%; *p* < 0.001) (Table S5). No clonal genotypes were detected in the dataset. STRUCTURE indicated that the optimal number of clusters was three (K = 3) (Table S6). All individuals were assigned primarily to one cluster regardless of where the population was found in the invasive range (i.e., core, early expansion, late expansion, invasion front). There was some evidence of population structure in the other two clusters; however, there was no clear geographic pattern (Figure 2). We also examined K = 4, which similarly revealed one major cluster for each individual and a minor one where there was some evidence of population structure. For example, four northern populations near the invasion front share the same minority cluster (Koochiching, St. Louis); this cluster is also shared with two populations in the range core (Figure 2).

**FIGURE 3:**
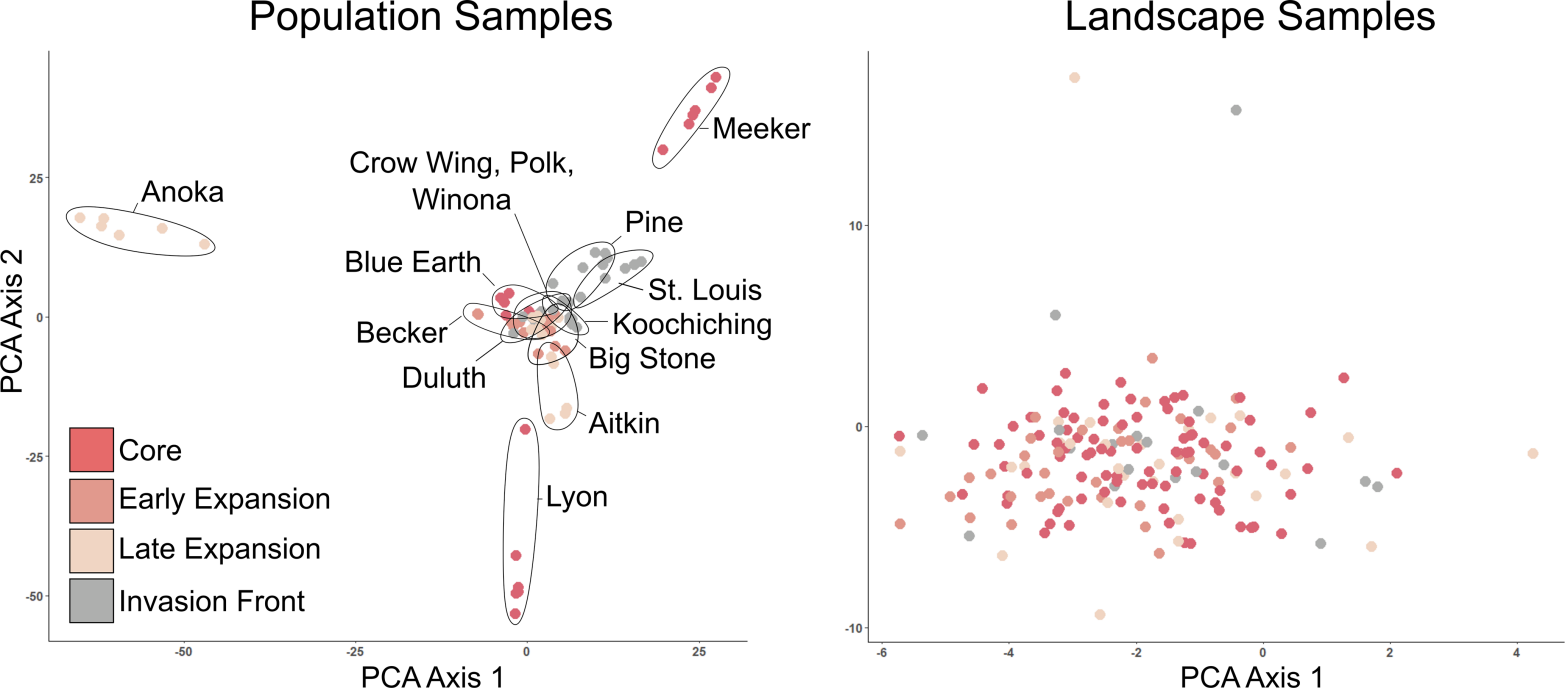
Principal components analysis (PCA) bi‐plots for population genomic data from 14 population samples (*n* = 6 per population) and 157 landscape samples (*n* = 1 per population).

The PCA did not indicate substantial population structure across the invaded range based on either population or landscape samples. For the population samples, there was some evidence of differentiation among three populations in or near the range core (Figure 3; PC1 and PC2 explained: 6.8% and 5.9% of variance, respectively). The PCA of landscape samples did not reveal a relationship between genetic similarity and geography over the time course of range expansion (Figure 3; PC1 and PC2 explained 1.7% and 1.3% of variance, respectively).

**FIGURE 4:**
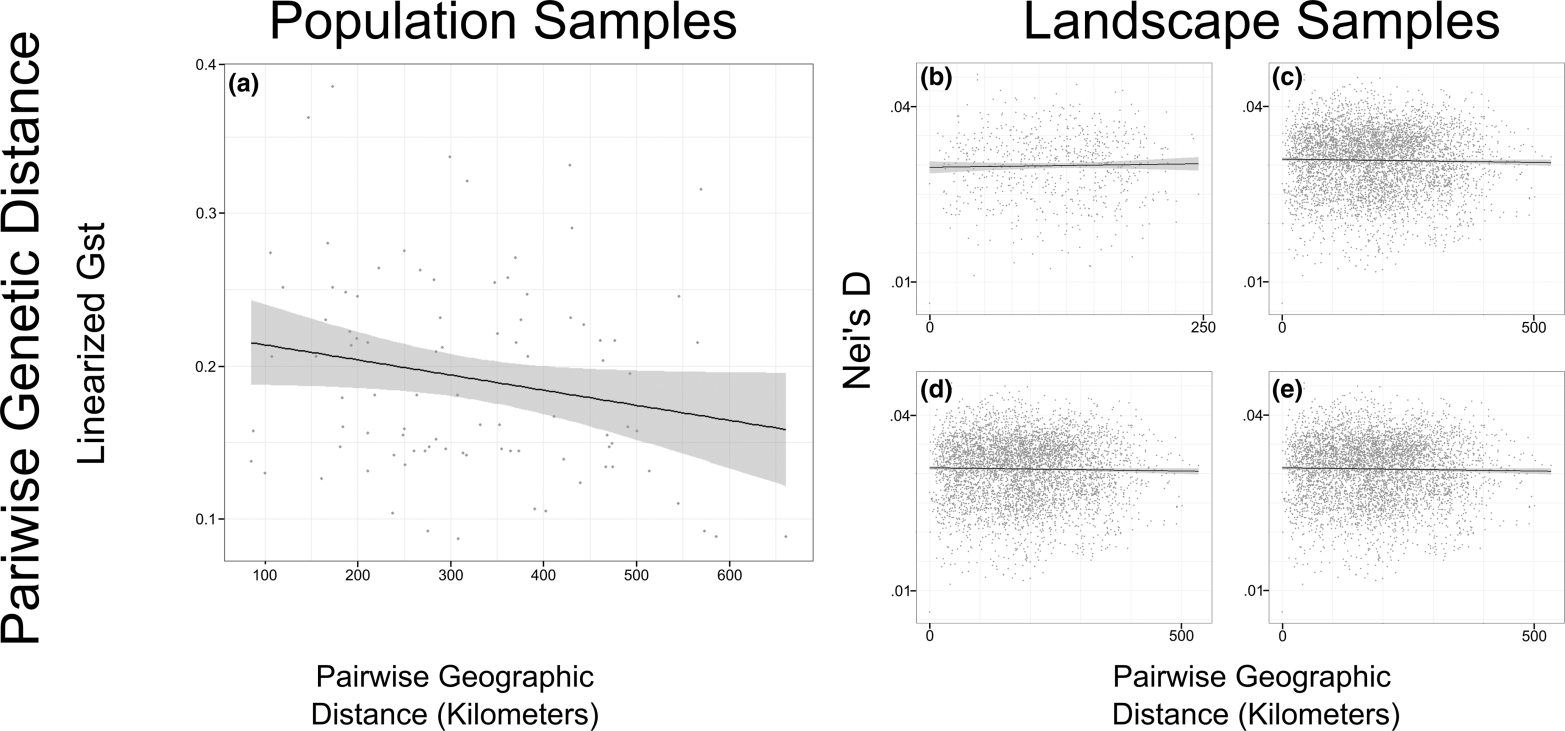
Isolation by distance (IBD) displayed as scatterplots of genetic distance versus geographic distance for a) population samples (a) and b–e) landscape samples (b–e). For landscape samples, we subset individuals for analyses by successive stages of invasion: b) range core (b), c) range core plus early expansion (c), d) range core, early, plus late expansion ranges (d), and e) all samples from across the four phases (e).

There was no evidence of IBD for either population (*R* ^*2*^ = 0.02; *p =* 0.418; Table 2; Table S7) or landscape samples (*R* ^*2*^ = 0.05; *p =* 0.676; Table S7). There was also no evidence of IBD when landscape samples were subset according to the four invasion phases that we defined (Figure 4).

**TABLE 2:** Pairwise estimates of genetic differentiation measured as G_st_ for 14 leafy spurge populations from four phases of range expansion (see Figure 1).

Core	Blue earth														
	Lyon	0.176													
	Meeker	0.215	0.266												
Early Expansion	Becker	0.126	0.173	0.208											
	Big Stone	0.125	0.171	0.209	0.112										
	Polk	0.118	0.171	0.205	0.115	0.119									
Late Expansion	Aitkin	0.124	0.187	0.216	0.126	0.127	0.127								
	Anoka	0.187	0.243	0.277	0.177	0.198	0.185	0.197							
	Crow Wing	0.134	0.188	0.219	0.116	0.128	0.126	0.136	0.201						
	Winona	0.084	0.134	0.181	0.081	0.084	0.081	0.095	0.153	0.096					
Invasion Front	Duluth	0.126	0.163	0.203	0.110	0.116	0.118	0.128	0.179	0.124	0.080				
	Koochiching	0.136	0.197	0.225	0.139	0.138	0.139	0.152	0.213	0.153	0.099	0.137			
	Pine	0.132	0.188	0.204	0.122	0.128	0.130	0.138	0.201	0.135	0.094	0.121	0.153		
	St. Louis	0.178	0.240	0.249	0.169	0.177	0.178	0.177	0.252	0.124	0.143	0.171	0.199	0.182	
		Blue Earth	Lyon	Meeker	Becker	Big Stone	Polk	Aitkin	Anoka	Crow Wing	Winona	Duluth	Koochiching	Pine	St. Louis

*Note*: Darker versus lighter shading of cells indicates higher versus lower values of pairwise G_st_.

Range expansion from the core area of invasion was accompanied by modest changes in genetic diversity. Heterozygosity declined from the core to the invasion front, as indicated by a significant interaction of latitude and longitude (*p =* 0.013; Table 3). However, the number of private alleles, Tajima's *D*, and the inbreeding coefficient did not change over the course of range expansion (Table 3). Estimates of N_e_ based on linkage disequilibrium using NeEstimator suggested that N_e_ was large (estimated at infinity) in all but three populations: Blue Earth (N_e_ = 134), Crow Wing (N_e_ = 210), and Koochiching (N_e_ = 17,836). Those three populations occur in the range core, late invasion, and invasion front portions of the range, respectively.

**TABLE 3:** ANOVA testing for a relationship between four metrics of sequence diversity and geography (latitude, longitude, and their interaction) for 14 population samples.

Model	Heterozygosity (Ho)			Private alleles			Tajima's *D*			Inbreeding (Gis)		
Term	Df	F	*p*	Df	F	*p*	Df	F	*p*	Df	F	*p*
Latitude	1	2.37	0.158	1	0.07	0.803	1	0.24	0.636	1	1.41	0.266
Longitude	1	0.48	0.506	1	1.29	0.285	1	1.22	0.298	1	0.04	0.846
Latitude × Longitude	1	9.49	**0.013**	1	0.84	0.382	1	0.02	0.887	1	0.48	0.508

*Note*: Bold indicates a *p*‐value less than 0.05.

### 3.2 Niche differentiation during range expansion

**FIGURE 5:**
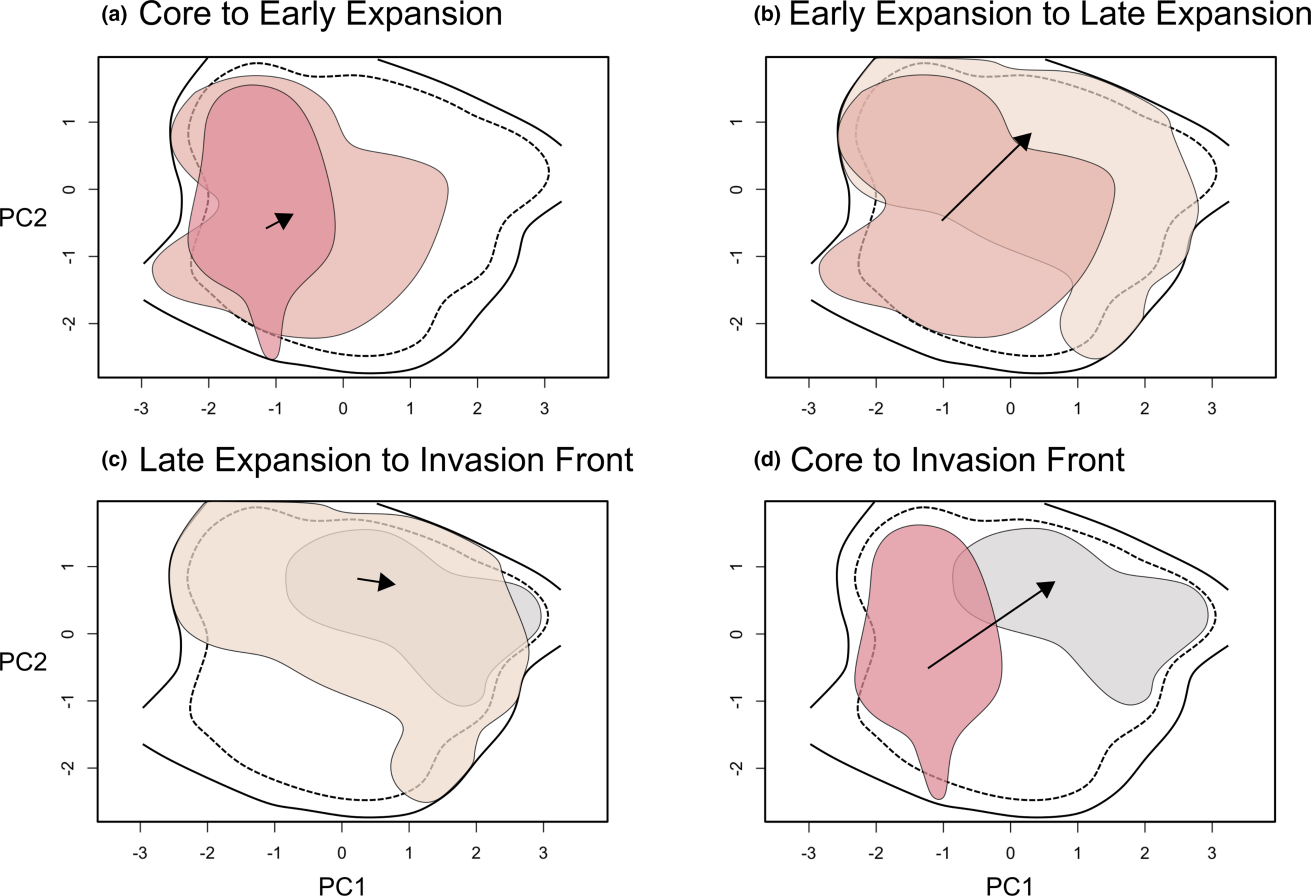
Niche overlap during range expansion in climate niche space. The extent of the background environment is outlined in black (solid = total niche space; dashed = 90% of extent). a) Core (dark pink) versus early expansion (light pink), b) Early expansion (light pink) versus late expansion (beige), c) Late expansion (beige) versus invasion front (gray), d) Core (dark pink) versus invasion front (gray). In all panels, the arrow represents the direction of shift in the centroid of niche space.

The climatic niche expanded during the invasion. Relative to the range core, the early expansion niche represented a sizable increase in niche breadth (Figure 5a; Table S8; overlap = 0.43; *p* < 0.01; expansion = 0.37; *p* < 0.01; stability = 0.63; *p* < 0.01). When comparing the early expansion and late expansion niches, there was similar evidence for a niche shift (Figure 5b; Table S8; overlap = 0.36, *p* < 0.01; expansion = 0.39, *p* < 0.01; stability = 0.61, *p* < 0.01). Between the late expansion niche and the invasion front, the null hypothesis of niche equivalency was not rejected (Table S8); the invasion front niche was contained within the late expansion niche (Figure 5c). When comparing the range core to the invasion front, there was near‐zero niche overlap (Figure 5d; Table S8; overlap = 0.03, *p* < 0.01; stability = 0.06, *p* < 0.01) and high expansion (Table S8; expansion = 0.94, *p* < 0.01). Niche differences between the core and invasion front were most apparent along environmental axes related to the temperature of the warmest quarter and the minimum temperature of the coldest month, rather than precipitation (Figure S6).

#### 3.2.1 Ecological niche model

**FIGURE 6:**
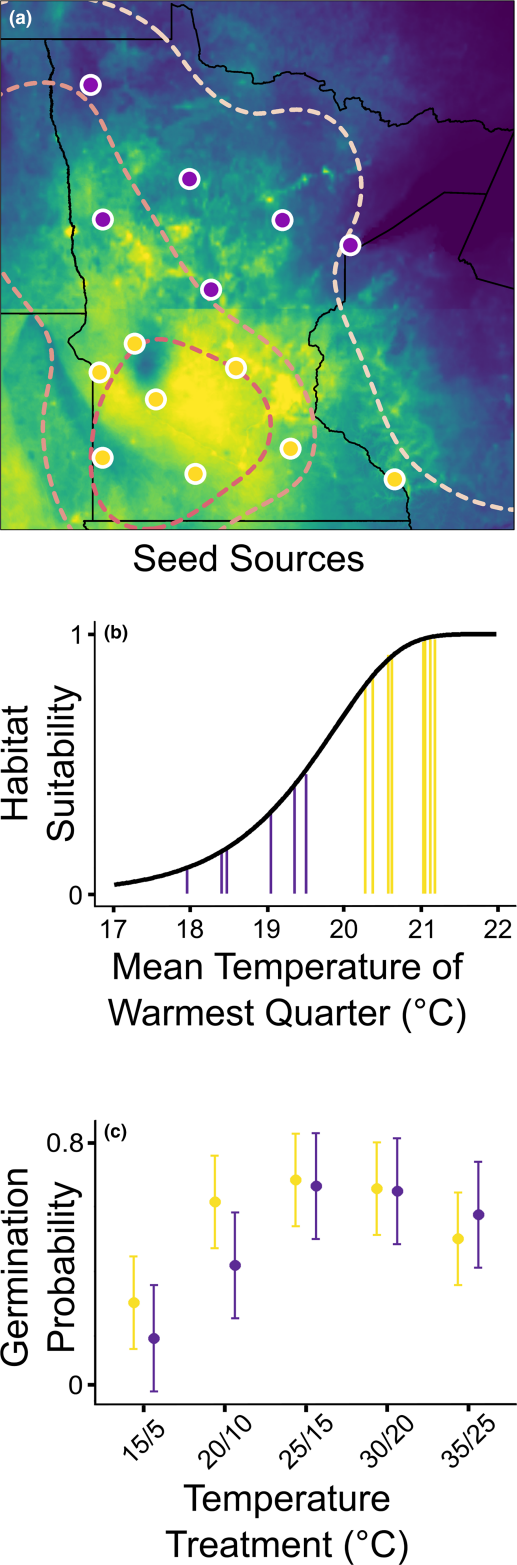
Ecological niche model (ENM) habitat suitability projections for seed source populations and germination probability for source regions. a) Habitat suitability projection from leafy spurge ENM. Predicted habitat suitability ranges from 0 (purple) to 1 (yellow). Dashed lines demarcate the chronosequence of range expansion. Seed source populations for the germination experiment are marked and colored yellow (>20°C) or purple (<20°C), depending on their mean temperature in the warmest quarter. b) Variable response curve for the mean temperature of the warmest quarter. Vertical yellow bars correspond to seed source populations from the warmer south, and vertical purple bars correspond to source populations from the cooler north. c) Mean germination probability (± SE) by temperature treatment (X axis) for seed source regions. The warmer southern region is shown in yellow, and the cooler northern region is shown in purple.

The model AUC (0.79) and sensitivity (0.75) metrics indicated moderately high discrimination and accuracy (Figure 6a). The variable response curves indicated the ENM was not overspecified (Figure S7). The mean temperature of the warmest quarter constituted the most important variable for the predicted probability of occurrence, hereafter “habitat suitability” (percent contribution = 81.5%; permutation importance = 67.8%). Warmer temperatures were associated with an increase in habitat suitability (*R* = 0.67; Figure S8). The minimum temperature of the coldest month had the second highest importance (percent contribution = 11.2%; permutation importance = 17.9%) and was modestly associated with increased habitat suitability (*R* = 0.31; Figure S8). Precipitation in the warmest quarter had the lowest importance (percent contribution = 7.3%; permutation importance = 14.3%) and was weakly correlated with habitat suitability (*R* = 0.02; Figure S8).

Populations from early versus late invasion were distinct along the mean temperature of the warmest quarter axis, with a disjunction at 20°C. This temperature also distinguished highly suitable habitats from less suitable habitats in the ENM (Figure 6a,b).

### 3.3 Evolution of germination behavior during range expansion

Populations from early versus late invasion responded differently to temperature regimes (source geographic region × temperature regime interaction: *p* = 0.003; Table S9; Figure 6c). The linear contrast between geographic regions for the 20/10°C treatment, the second coldest treatment, was marginally significant (*p* = 0.09); contrasts between regions for all other temperature treatments were not significant (*p* > 0.77; Table S10). Germination probability increased with temperature for both geographic regions and plateaued (*p* < 0.001; Table S9; Figure 6c).

## 4 DISCUSSION

Rapid evolution is increasingly recognized as an important process contributing to range expansion and influencing forecasts of future invasion (Clements & Jones, 2021; Prentis et al., 2008; van Boheemen et al., 2019). However, our understanding of the temporal and spatial scale over which niche and trait divergence contribute to invasion at leading range edges remains unresolved. Our study took advantage of a well‐documented invasion history to synthesize the consequences of recent range expansion for population genomic diversity, niche breadth, and germination behavior, a trait important in the colonization of new habitats. We found that leafy spurge populations experienced only modest losses in sequence diversity over the chronosequence of invasion and that recruitment within invaded populations occurred via seeds rather than strictly from clonal expansion. Range expansion involved climatic niche expansion, and ENMs suggested that the mean temperature of the warmest quarter had the strongest influence on habitat suitability. Populations differentiated in germination behavior in response to temperature, where leading‐edge populations exhibited a trend of increased dormancy at low temperatures. Our results suggest that evolution during range expansion may be important to consider in the development of models forecasting range shifts in current and future climates.

Loss of genetic diversity during range expansion may have fitness consequences and limit adaptive capacity (Dlugosch & Parker, 2008; Lee, 2002). The magnitude of within‐population diversity is also potentially important for the success of eradication measures, which may not be effective against all genotypes. We found that range expansion was only accompanied by small losses in heterozygosity but no changes in the inbreeding coefficient or the number of private alleles. We also did not observe evidence of genetic bottlenecks based on linkage disequilibrium. We identified some divergence among populations in or near the range core, while others occupied intermediate positions in PCA space. Prevalent long‐distance dispersal from the expanding core to the invasion front could reintroduce allelic variation lost due to bottlenecks in the colonization process. Additionally, admixture among independently introduced populations (i.e., invasion fronts not included in our analyses) could contribute to within‐population genetic diversity, especially when there is genetic divergence between sources of introduction (Gaskin & Schaal, 2002; Gibson et al., 2021; Keller & Taylor, 2010). Although we do not observe novel genotypes or elevated numbers of private alleles at the periphery of our focal region, which would be indicative of admixture between invasion fronts, more work is needed to evaluate this potential influence.

Regardless of the mechanism, the extent of within‐population variation may have important implications for eradication and management. Although leafy spurge can spread clonally, it does not appear that populations constitute one or a few clones that spread widely upon colonization. Instead, recruitment from seeds contributes substantially to population growth. This finding has implications for integrated pest management (IPM) strategies. Management techniques that reduce above‐ground productivity and seed production may be important in addition to those that reduce clonal spread by disturbing root systems (e.g., biocontrol agents that feed primarily on roots; Hanson et al. 1997). In addition to natural disturbance, improperly timed grazing or herbicide applications could create open spaces for recruitment from seeds and inadvertently increase the density and abundance of the invader (Gaskin et al., 2022). As seeds may remain dormant for as long as 5 years (Selleck et al., 1962), seed banks may further complicate control efforts. The combination of recruitment from seeds and clonal growth contributes to the population dynamics of leafy spurge, and therefore, multiple eradication approaches may be necessary to cause local population extinction.

In addition to gene flow during invasion, polyploidy may be an important factor influencing losses of genetic variation during colonization bottlenecks and the capacity for range expansion. Polyploids often maintain higher levels of genetic variation (Otto & Whitton, 2000), and there is some evidence that phenotypic plasticity is greater in synthetically produced autopolyploids (Mattingly & Hovick, 2023). These factors have been used to explain why polyploids may be better invaders than diploids (Pandit et al., 2011). Leafy spurge is an auto‐allohexaploid, suggesting that its higher N_e_ should facilitate the maintenance of genetic diversity within populations and minimize divergence among them. Polyploidy has also been suggested to increase the capacity for adaptive evolution (Otto & Whitton, 2000), which could also contribute to success as an invader. It is important to recognize that losses of diversity in DNA sequences do not necessarily translate to losses of variation in quantitative traits (Reed & Frankham, 2001). Nevertheless, it is possible that polyploidy contributed to rapid invasion in leafy spurge, but more work is needed to distinguish the contribution of polyploidy from other factors.

Accumulating evidence suggests that invasive plant species frequently undergo climatic niche shifts during range expansion (Medley, 2010; Atwater et al., 2018; van Boheemen et al., 2019; Bates & Bertelsmeier, 2021; but see Petitpierre et al., 2012; Liu et al., 2020). Such niche shifts can complicate the reliability of models that seek to predict future invasions, as these models generally assume niche conservation. Consistent with past findings, we observed climatic niche expansion throughout most of the range expansion, except from the late expansion region to the invasion front. From an ENM, we found that warm‐season temperature had the strongest influence on habitat suitability and therefore may be one source of divergent selection from the range core to the invasion front. Of course, climate change has already caused poleward shifts in plant species distributions through climatic niche matching (Clements & Ditommaso, 2011; Parmesan, 2006; Parmesan & Hanley, 2015). Therefore, some range expansion may simply involve dispersal to already climatically suitable habitats. However, adaptation may be necessary for continued range expansion, especially where the species is already at its climatic niche limit (Clements & Ditommaso, 2011). In leafy spurge, minimal range expansion northward has been observed in the last 30+ years and populations remain small/low density at the invasion front. In addition, ENMs suggest that invasion‐front populations have very low habitat suitability in terms of climate. Overall, our results are inconsistent with the hypothesis that the leading edge is suitable but expansion is limited by dispersal. Instead, responses to divergent selection may be important for persistence at the leading range edge and for further range expansion. These findings also hold implications for management approaches: recognizing and accounting for niche shifts could improve the accuracy of distribution models for forecasting future invasions and, by extension, help optimize resource allocation for eradication efforts. Integrating knowledge of which environmental factors underlie niche expansion will be key to designing more effective management strategies that not only suppress existing populations but also anticipate future invasions.

Divergence in trait expression at a leading range edge can be driven by local adaptation, phenotypic plasticity, and/or maternal environmental effects (Des Roches et al., 2017; Westerband et al., 2021). Phenotypic plasticity is considered important during the early stages of invasion because it allows introduced populations to establish themselves in a broader range of environmental conditions (Funk, 2008; Lande, 2015; Richards et al., 2006; Sexton et al., 2002). In germination traits, plasticity could represent a means of habitat selection and niche construction (Donohue, 2003, 2005), by which leafy spurge in the leading edge germinates optimally at the onset of spring conditions (warmer temperatures in northern latitudes). Likewise, rapid evolution during range expansion can result from selection on loci that influence dormancy and/or germination timing (Clements & Ditommaso, 2011; Clements & Jones, 2021; Hodgins et al., 2018). We found a trend of increased dormancy at lower temperatures in leading edge populations (*p* = 0.09 for 20/10°C treatment). One possibility is that germination at colder temperatures exposes seedlings to more unpredictable environments (e.g., late‐season frost) and thus that selection favors germination later in the season for leafy spurge at its northern range limit. Interestingly, other work has suggested that reduced dormancy evolves at leading range edges (Tabassum & Leishman, 2018), contrary to our findings. We cannot exclude the possibility that maternal environmental effects influenced our estimates of germination probability because we used field‐collected seeds. However, our findings are contrary to what we would have expected if maternal environmental effects had been important: we would have expected higher germination for leading‐edge populations in cold treatments because those populations come from colder environments. Although warm‐season temperature is most strongly associated with habitat suitability and relevant to germination in leafy spurge, it is also possible that other (correlated) variables influenced the evolution of germination behavior. Overall, our results suggest that range expansion involved niche expansion and potentially the evolution of germination timing, which may have been important in establishment at the leading range edge.

Early life‐history transitions are thought to be under strong selection because of their cascading effects on later life stages (Baskin & Baskin, 1971; Donohue, 2002, 2005; Marks & Prince, 1981). In plant populations, environmental conditions at the time of germination can alter the strength and direction of natural selection on postgermination traits (D'Aguillo & Donohue, 2023; Donohue et al., 2010). In turn, this can affect the competitive environment, resource availability, and density‐dependent selective agents experienced by populations (Donohue et al., 2010). While we did not investigate postgermination traits, it would be valuable to determine whether germination timing influences performance at later life‐history stages (e.g., fecundity), especially in leading‐edge populations. Knowledge of germination timing could help identify optimal windows for applying herbicides or biocontrol agents. If germination timing or success is found to affect performance at later life stages, management strategies could aim to destabilize these windows of opportunity, potentially preventing the establishment of new invasive populations and limiting the growth and persistence of existing ones.

Our results suggest that even over the course of a fairly rapid invasion, losses of genomic variation may be minimal. In leafy spurge, this may have occurred because of substantial gene flow during invasion and/or polyploidy. Higher levels of genetic variation can challenge management when genotypes vary in their responses to eradication measures (Gaskin et al., 2020). We also found that trait divergence may have contributed to climatic niche expansion and, thus, to the spatial extent of invasion. Forecasts of continued invasion typically rely on species distribution models (SDMs), which rarely take into account evolution. As such, models may fail to predict the complete extent of range expansion or the severity of range infilling. Evolution‐free SDMs are likely still valuable for management planning over meaningful spatial and temporal scales in many systems. However, in systems where local adaptation is extensive, forecasts of range shifts with climate change may require the construction of regional SDMs that account for evolution.

## FUNDING INFORMATION

Funding for this project was provided by the Minnesota Invasive Terrestrial Plants and Pests Center through the Environment and Natural Resources Trust Fund as recommended by the Legislative‐Citizen Commission on Minnesota Resources (LCCMR). TAL was supported by the University of Minnesota Doctoral Dissertation Fellowship.

## CONFLICT OF INTEREST STATEMENT

The authors have no relevant financial or non‐financial interests to disclose.

## Supporting information


Data S1.

## Data Availability

All DNA sequencing data are archived and available through DOI: 10.5061/dryad.kd51c5bch

## References

[eva13593-bib-0001] Araújo, M. B. , Anderson, R. P. , Márcia Barbosa, A. , Beale, C. M. , Dormann, C. F. , Early, R. , Garcia, R. A. , Guisan, A. , Maiorano, L. , Naimi, B. , O'Hara, R. B. , Zimmermann, N. E. , & Rahbek, C. (2019). Standards for distribution models in biodiversity assessments. Science Advances, 5, eaat4858. 30746437 10.1126/sciadv.aat4858 PMC6357756

[eva13593-bib-0002] Atwater, D. Z. , Ervine, C. , & Barney, J. N. (2018). Climatic niche shifts are common in introduced plants. Nature Ecology & Evolution, 2, 34–43. 29203919 10.1038/s41559-017-0396-z

[eva13593-bib-0003] Austerlitz, F. , Jung‐Muller, B. , Godelle, B. , & Gouyon, P.‐H. (1997). Evolution of coalescence times, genetic diversity and structure during colonization. Theoretical Population Biology, 51, 148–164.

[eva13593-bib-0004] Bakke, A. L. (1936). Leafy spurge, Euphorbia esula L. Iowa Agricultural Experiment Station, 189, 209–245.

[eva13593-bib-0005] Baniaga, A. E. , Marx, H. E. , Arrigo, N. , & Barker, M. S. (2020). Polyploid plants have faster rates of multivariate niche differentiation than their diploid relatives. Ecology Letters, 23, 68–78. 31637845 10.1111/ele.13402

[eva13593-bib-0006] Baskin, J. M. , & Baskin, C. C. (1971). Germination of winter annuals in July and survival of the seedlings. Bulletin of the Torrey Botanical Club, 98, 272–276.

[eva13593-bib-0007] Bates, O. K. , & Bertelsmeier, C. (2021). Climatic niche shifts in introduced species. Current Biology, 31, R1252–R1266. 34637738 10.1016/j.cub.2021.08.035

[eva13593-bib-0008] Batho, G. (1932). Leafy spurge (p. 106). Department of Agriculture and Immigration Winnipeg.

[eva13593-bib-0009] Blaine Marchant, D. , Soltis, D. E. , & Soltis, P. S. (2016). Patterns of abiotic niche shifts in allopolyploids relative to their progenitors. The New Phytologist, 212, 708–718. 27399976 10.1111/nph.14069

[eva13593-bib-0010] Bolger, A. M. , Lohse, M. , & Usadel, B. (2014). Trimmomatic: A flexible trimmer for Illumina sequence data. Bioinformatics, 30, 2114–2120. 24695404 10.1093/bioinformatics/btu170 PMC4103590

[eva13593-bib-0011] Brittingham, H. A. , Koski, M. H. , & Ashman, T.‐L. (2018). Higher ploidy is associated with reduced range breadth in the Potentilleae tribe. American Journal of Botany, 105, 700–710. 29608209 10.1002/ajb2.1046

[eva13593-bib-0012] Brochmann, C. , Brysting, A. K. , Alsos, I. G. , Borgen, L. , Grundt, H. H. , Scheen, A. C. , & Elven, R. (2004). Polyploidy in arctic plants. Biological journal of the Linnean society, 82, 521–536.

[eva13593-bib-0013] Capblancq, T. , Fitzpatrick, M. C. , Bay, R. A. , Exposito‐Alonso, M. , & Keller, S. R. (2020). Genomic prediction of (mal)adaptation across current and future climatic landscapes. Annual Review of Ecology, Evolution, and Systematics, 51, 245–269.

[eva13593-bib-0014] Chao, W. S. , Horvath, D. P. , Anderson, J. V. , & Foley, M. E. (2005). Potential model weeds to study genomics, ecology, and physiology in the 21st century. Weed Science, 53, 929–937.

[eva13593-bib-0015] Chapman, D. S. , Scalone, R. , Štefanić, E. , & Bullock, J. M. (2017). Mechanistic species distribution modeling reveals a niche shift during invasion. Ecology, 98, 1671–1680. 28369815 10.1002/ecy.1835

[eva13593-bib-0016] Chardon, N. I. , Pironon, S. , Peterson, M. L. , & Doak, D. F. (2020). Incorporating intraspecific variation into species distribution models improves distribution predictions, but cannot predict species traits for a wide‐spread plant species. Ecography, 43, 60–74.

[eva13593-bib-0017] Chown, S. L. , Hodgins, K. A. , Griffin, P. C. , Oakeshott, J. G. , Byrne, M. , & Hoffmann, A. A. (2015). Biological invasions, climate change and genomics. Evolutionary Applications, 8, 23–46. 25667601 10.1111/eva.12234 PMC4310580

[eva13593-bib-0018] Clark, L. V. , Lipka, A. E. , & Sacks, E. J. (2019). polyRAD: Genotype calling with uncertainty from sequencing data in Polyploids and diploids. G3: Genes, Genomes, Genetics, G3 9, 663–673. 10.1534/g3.118.200913 PMC6404598 30655271

[eva13593-bib-0019] Clark, L. V. , Mays, W. , Lipka, A. E. , & Sacks, E. J. (2022). A population‐level statistic for assessing Mendelian behavior of genotyping‐by‐sequencing data from highly duplicated genomes. BMC Bioinformatics, 23, 101. 35317727 10.1186/s12859-022-04635-9 PMC8939213

[eva13593-bib-0020] Clements, D. R. , & Ditommaso, A. (2011). Climate change and weed adaptation: Can evolution of invasive plants lead to greater range expansion than forecasted? Weed Research, 51, 227–240.

[eva13593-bib-0021] Clements, D. R. , & Jones, V. L. (2021). Rapid evolution of invasive weeds under climate change: Present evidence and future research needs. Frontiers in Agronomy, 3, 664034.

[eva13593-bib-0022] Colautti, R. I. , & Barrett, S. C. H. (2013). Rapid adaptation to climate facilitates range expansion of an invasive plant. Science, 342, 364–366. 24136968 10.1126/science.1242121

[eva13593-bib-0023] Colautti, R. I. , & Lau, J. A. (2015). Contemporary evolution during invasion: Evidence for differentiation, natural selection, and local adaptation. Molecular Ecology, 24, 1999–2017. 25891044 10.1111/mec.13162

[eva13593-bib-0024] Collart, F. , Hedenäs, L. , Broennimann, O. , Guisan, A. , & Vanderpoorten, A. (2020). Intraspecific differentiation: Implications for niche and distribution modelling. Journal of Biogeography, 48, 415–426.

[eva13593-bib-0025] D'Aguillo, M. , & Donohue, K. (2023). Changes in phenology can alter patterns of natural selection: The joint evolution of germination time and postgermination traits. The New Phytologist, 238, 405–421. 10.1111/nph.18711 36600403

[eva13593-bib-0026] Des Roches, S. , Post, D. M. , Turley, N. E. , Bailey, J. K. , Hendry, A. P. , Kinnison, M. T. , Schweitzer, J. A. , & Palkovacs, E. P. (2018). The ecological importance of intraspecific variation. Nature Ecology & Evolution, 2, 57–64. 29203921 10.1038/s41559-017-0402-5

[eva13593-bib-0027] Di Cola, V. , Broennimann, O. , Petitpierre, B. , Breiner, F. T. , d'Amen, M. , Randin, C. , Engler, R. , Pottier, J. , Pio, D. , Dubuis, A. , & Pellissier, L. (2017). Ecospat: An R package to support spatial analyses and modeling of species niches and distributions. Ecography, 40, 774–787.

[eva13593-bib-0028] Dixon, A. L. , & Busch, J. W. (2017). Common garden test of range limits as predicted by a species distribution model in the annual plant Mimulus bicolor. American Journal of Botany, 104, 817–827. 28645920 10.3732/ajb.1600414

[eva13593-bib-0029] Dlugosch, K. M. , & Parker, I. M. (2008). Founding events in species invasions: Genetic variation, adaptive evolution, and the role of multiple introductions. Molecular Ecology, 17, 431–449. 17908213 10.1111/j.1365-294X.2007.03538.x

[eva13593-bib-0030] Do, C. , Waples, R. S. , Peel, D. , Macbeth, G. M. , Tillett, B. J. , & Ovenden, J. R. (2014). NeEstimator v2: re‐implementation of software for the estimation of contemporary effective population size (Ne ) from genetic data. Mol Ecol Resour, 14(1), 209–214. 23992227 10.1111/1755-0998.12157

[eva13593-bib-0031] Donohue, K. (2002). Germination timing influences natural selection on life‐history characters in Arabidopsis Thaliana. Ecology, 83, 1006–1016.

[eva13593-bib-0032] Donohue, K. (2003). Setting the stage: Phenotypic plasticity as habitat selection. International Journal of Plant Sciences, 164, S79–S92.

[eva13593-bib-0033] Donohue, K. (2005). Niche construction through phenological plasticity: Life history dynamics and ecological consequences. The New Phytologist, 166, 83–92. 15760353 10.1111/j.1469-8137.2005.01357.x

[eva13593-bib-0034] Donohue, K. , Rubio de Casas, R. , Burghardt, L. , Kovach, K. , & Willis, C. G. (2010). Germination, postgermination adaptation, and species ecological ranges. Annual Review of Ecology, Evolution, and Systematics, 41, 293–319.

[eva13593-bib-0035] Dufresne, F. , Stift, M. , Vergilino, R. , & Mable, B. K. (2014). Recent progress and challenges in population genetics of polyploid organisms: An overview of current state‐of‐the‐art molecular and statistical tools. Molecular Ecology, 23, 40–69. 24188632 10.1111/mec.12581

[eva13593-bib-0036] Duncan, C. A. , Jachetta, J. J. , Brown, M. L. , Carrithers, V. F. , Clark, J. K. , Ditomaso, J. M. , Lym, R. G. , Mcdaniel, K. C. , Renz, M. J. , & Rice, P. M. (2004). Assessing the economic, environmental, and societal losses from invasive plants on rangeland and wildlands. Weed Technology, 18, 1411–1416.

[eva13593-bib-0037] Dunn, P. H. (1979). The distribution of leafy spurge (Euphorbia esula) and other weedy euphorbia spp. in the United States. Weed Science, 27, 509–516.

[eva13593-bib-0038] Dunn, P. H. (1985). Origins of leafy spurge in North America. In A. K. Watson (Ed.), Leafy spurge, monograph No. 3 (pp. 7–13). Weed Science Society of America.

[eva13593-bib-0039] Earl, D. A. , & VonHoldt, B. M. (2012). STRUCTURE HARVESTER: A website and program for visualizing STRUCTURE output and implementing the Evanno method. Conservation Genetics Resources, 4, 359–361.

[eva13593-bib-0040] Elith, J. , & Leathwick, J. R. (2009). Species distribution models: Ecological explanation and prediction across space and time. Annual Review of Ecology, Evolution, and Systematics, 40, 677–697.

[eva13593-bib-0151] ESRI . (2022). *ArcGIS Pro: Release 3.1.0*. Environmental Systems Research Institute.

[eva13593-bib-0041] Evanno, G. , Regnaut, S. , & Goudet, J. (2005). Detecting the number of clusters of individuals using the software STRUCTURE: A simulation study. Molecular Ecology, 14, 2611–2620. 15969739 10.1111/j.1365-294X.2005.02553.x

[eva13593-bib-0042] Excoffier, L. (2004). Patterns of DNA sequence diversity and genetic structure after a range expansion: Lessons from the infinite‐Island model. Molecular Ecology, 13, 853–864. 15012760 10.1046/j.1365-294x.2003.02004.x

[eva13593-bib-0043] Excoffier, L. , & Ray, N. (2008). Surfing during population expansions promotes genetic revolutions and structuration. Trends in Ecology & Evolution, 23, 347–351. 18502536 10.1016/j.tree.2008.04.004

[eva13593-bib-0044] Excoffier, L. , Smouse, P. E. , & Quattro, J. M. (1992). Analysis of molecular variance inferred from metric distances among DNA haplotypes: Application to human mitochondrial DNA restriction data. Genetics, 131, 479–491. 1644282 10.1093/genetics/131.2.479 PMC1205020

[eva13593-bib-0152] Fox, J. , & Weisberg, S. (2019). An R Companion to Applied Regression (Third ed.). Sage. https://socialsciences.mcmaster.ca/jfox/Books/Companion/

[eva13593-bib-0046] Funk, J. L. (2008). Differences in plasticity between invasive and native plants from a low resource environment. Journal of Ecology, 96, 1162–1173.

[eva13593-bib-0047] Gaskin, J. F. , Espeland, E. , Johnson, C. D. , Larson, D. L. , Mangold, J. M. , McGee, R. A. , Milner, C. , Paudel, S. , Pearson, D. E. , Perkins, L. B. , Prosser, C. W. , Runyon, J. B. , Sing, S. E. , Sylvain, Z. A. , Symstad, A. J. , & Tekiela, D. R. (2020). Managing invasive plants on Great Plains grasslands: A discussion of current challenges. Rangeland Ecology & Management, 78, 235–249. 10.1016/j.rama.2020.04.003

[eva13593-bib-0048] Gaskin, J. F. , Littlefield, J. L. , Rand, T. A. , & West, N. M. (2022). Variation in reproductive mode across the latitudinal range of invasive Russian knapweed. AoB Plants, 14, lac032. 10.1093/aobpla/plac032 PMC9346633 35937548

[eva13593-bib-0049] Gaskin, J. F. , & Schaal, B. A. (2002). Hybrid *Tamarix* widespread in U.S. invasion and undetected in native Asian range. Proceedings of the National Academy of Sciences of the United States of America, 99, 11256–11259. 12177412 10.1073/pnas.132403299 PMC123243

[eva13593-bib-0050] Gerard, D. , & Ferrão, L. F. V. (2020). Priors for genotyping polyploids. Bioinformatics, 36, 1795–1800. 32176767 10.1093/bioinformatics/btz852

[eva13593-bib-0051] Gibson, M. J. , Torres, M. d. L. , Brandvain, Y. , & Moyle, L. C. (2021). Introgression shapes fruit color convergence in invasive Galápagos tomato. eLife, 10, e64165. 10.7554/eLife.64165 34165082 PMC8294854

[eva13593-bib-0052] Glennon, K. L. , Ritchie, M. E. , & Segraves, K. A. (2014). Evidence for shared broad‐scale climatic niches of diploid and polyploid plants. Ecology Letters, 17, 574–582. 24818236 10.1111/ele.12259

[eva13593-bib-0053] Goodsman, D. W. , Cooke, B. , Coltman, D. W. , & Lewis, M. A. (2014). The genetic signature of rapid range expansions: How dispersal, growth and invasion speed impact heterozygosity and allele surfing. Theoretical Population Biology, 98, 1–10. 25201435 10.1016/j.tpb.2014.08.005

[eva13593-bib-0054] Gorton, A. J. , Tiffin, P. , & Moeller, D. A. (2019). Does adaptation to historical climate shape plant responses to future rainfall patterns? A rainfall manipulation experiment with common ragweed. Oecologia, 190, 941–953. 31289920 10.1007/s00442-019-04463-4

[eva13593-bib-0154] Hansen, R. W. , Richard, R. D. , Parker, P. E. , & Wendell, L. E. (1997). Distribution of biological control agents of leafy spurge (Euphorbia esula L.) in the United States: 1988–1996. Biological Control, 10, 129–142.

[eva13593-bib-0055] Hanson, H. C. , & Rudd, V. E. (1933). Leafy spurge: Life history and habits. North Dakota Agricultural College Experiment Station Bulletin, 266, 1–24.

[eva13593-bib-0056] Hein, D. G. , & Miller, S. D. (1992). Influence of leafy spurge on forage utilization by cattle. Journal of Range Management, 45, 405.

[eva13593-bib-0057] Hijmans, R. , Phillips, S. , Leathwick, J. , & Elith, J. (2022). dismo: Species distribution modeling. Version 1.3‐9 https://cran.r‐project.org/web/packages/dismo/index.html

[eva13593-bib-0058] Hodgins, K. A. , Bock, D. G. , & Rieseberg, L. H. (2018). Trait evolution in invasive species. Annual Plant Reviews Online, 1, 459–496.

[eva13593-bib-0059] Horvath, D. , Anderson, J. V. , Chao, W. S. , Foley, M. E. , & Doğramaci, M. (2015). Leafy spurge genomics: A model perennial weed to investigate development, stress responses, and invasiveness. In G. Sablok , S. Kumar , S. Ueno , J. Kuo , & C. Varotto (Eds.), Advances in the understanding of biological sciences using next generation sequencing (NGS) approaches (pp. 63–78). Springer International Publishing.

[eva13593-bib-0060] Horvath, D. P. , Patel, S. , Doğramaci, M. , Chao, W. S. , Anderson, J. V. , Foley, M. E. , Scheffler, B. , Lazo, G. , Dorn, K. , Yan, C. , Childers, A. , Schatz, M. , & Marcus, S. (2018). Gene space and transcriptome assemblies of leafy spurge (Euphorbia esula) identify promoter sequences, repetitive elements, high‐quality markers, and a full‐length chloroplast genome. Weed Science, 66, 355–367.

[eva13593-bib-0061] Hutchison, D. W. , & Templeton, A. R. (1999). Correlation of pairwise genetic and geographic distance measures: Inferring the relative influences of gene flow and drift on the distribution of genetic variability. Evolution, 53, 1898–1914. 28565459 10.1111/j.1558-5646.1999.tb04571.x

[eva13593-bib-0062] Jombart, T. (2008). Adegenet: A R package for the multivariate analysis of genetic markers. Bioinformatics, 24, 1403–1405. 18397895 10.1093/bioinformatics/btn129

[eva13593-bib-0063] Kamvar, Z. N. , Brooks, J. C. , & Grünwald, N. J. (2015). Novel R tools for analysis of genome‐wide population genetic data with emphasis on clonality. Frontiers in Genetics, 6, 208. 26113860 10.3389/fgene.2015.00208 PMC4462096

[eva13593-bib-0064] Keller, S. R. , & Taylor, D. R. (2008). History, chance and adaptation during biological invasion: Separating stochastic phenotypic evolution from response to selection. Ecology Letters, 11, 852–866. 18422638 10.1111/j.1461-0248.2008.01188.x

[eva13593-bib-0065] Keller, S. R. , & Taylor, D. R. (2010). Genomic admixture increases fitness during a biological invasion. Journal of Evolutionary Biology, 23, 1720–1731. 20626546 10.1111/j.1420-9101.2010.02037.x

[eva13593-bib-0066] Klopfstein, S. , Currat, M. , & Excoffier, L. (2006). The fate of mutations surfing on the wave of a range expansion. Molecular Biology and Evolution, 23, 482–490. 16280540 10.1093/molbev/msj057

[eva13593-bib-0067] Koski, M. H. , Layman, N. C. , Prior, C. J. , Busch, J. W. , & Galloway, L. F. (2019). Selfing ability and drift load evolve with range expansion. Evolution Letters, 3, 500–512. 31636942 10.1002/evl3.136 PMC6791181

[eva13593-bib-0068] Lacey, J. R. , Wallander, R. , & Olson‐Rutz, K. (1992). Recovery, germinability, and viability of leafy spurge (Euphorbia esula) seeds ingested by sheep and goats. Weed Technology, 6, 599–602.

[eva13593-bib-0069] Lachmuth, S. , Durka, W. , & Schurr, F. M. (2010). The making of a rapid plant invader: Genetic diversity and differentiation in the native and invaded range of Senecio inaequidens. Molecular Ecology, 19, 3952–3967. 20854275 10.1111/j.1365-294X.2010.04797.x

[eva13593-bib-0070] Lake, T. A. , Briscoe Runquist, R. D. , & Moeller, D. A. (2020). Predicting range expansion of invasive species: Pitfalls and best practices for obtaining biologically realistic projections. Diversity and Distributions, 24, 348–1779.

[eva13593-bib-0071] Lakela, O. (1965). A Flora of northeastern Minnesota, NED‐New edition. University of Minnesota Press.

[eva13593-bib-0072] Lande, R. (2015). Evolution of phenotypic plasticity in colonizing species. Molecular Ecology, 24, 2038–2045. 25558898 10.1111/mec.13037

[eva13593-bib-0073] Lee, C. E. (2002). Evolutionary genetics of invasive species. Trends in Ecology & Evolution, 17, 386–391.

[eva13593-bib-0074] Lehe, R. , Hallatschek, O. , & Peliti, L. (2012). The rate of beneficial mutations surfing on the wave of a range expansion. PLoS Computational Biology, 8, e1002447. 22479175 10.1371/journal.pcbi.1002447 PMC3315454

[eva13593-bib-0075] Liu, C. , Wolter, C. , Xian, W. , & Jeschke, J. M. (2020). Most invasive species largely conserve their climatic niche. Proceedings of the National Academy of Sciences of the United States of America, 117, 23643–23651. 32883880 10.1073/pnas.2004289117 PMC7519298

[eva13593-bib-0076] Lym, R. G. (1998). The biology and integrated management of leafy spurge (Euphorbia esula) on North Dakota rangeland. Weed Technology, 12, 367–373.

[eva13593-bib-0078] Lym, R. G. , & Kirby, D. R. (1987). Cattle foraging behavior in leafy spurge (Euphorbia esula)‐infested rangeland. Weed Technology, 1, 314–318.

[eva13593-bib-0079] Ma, L. , Cao, L.‐J. , Hoffmann, A. A. , Gong, Y. J. , Chen, J. C. , Chen, H. S. , Wang, X. B. , Zeng, A. P. , Wei, S. J. , & Zhou, Z. S. (2020). Rapid and strong population genetic differentiation and genomic signatures of climatic adaptation in an invasive mealybug. Diversity and Distributions, 26, 610–622.

[eva13593-bib-0080] Marks, M. , & Prince, S. (1981). Influence of germination date on survival and fecundity in wild lettuce Lactuca Serriola. Oikos, 36, 326–330.

[eva13593-bib-0081] Mathias, A. , & Kisdi, E. (2002). Adaptive diversification of germination strategies. Proceedings of the Biological Sciences, 269, 151–155. 10.1098/rspb.2001.1867 PMC1690878 11798430

[eva13593-bib-0082] Mattingly, K. Z. , & Hovick, S. M. (2023). Autopolyploids of Arabidopsis thaliana are more phenotypically plastic than their diploid progenitors. Annals of Botany, 131, 45–58. 34175922 10.1093/aob/mcab081 PMC9904351

[eva13593-bib-0083] Medley, K. A. (2010). Niche shifts during the global invasion of the Asian tiger mosquito, Aedes albopictus Skuse (Culicidae), revealed by reciprocal distribution models. Global Ecology and Biogeography, 19, 122–133.

[eva13593-bib-0084] Meirmans, P. G. (2012). AMOVA‐based clustering of population genetic data. The Journal of Heredity, 103, 744–750. 22896561 10.1093/jhered/ess047

[eva13593-bib-0085] Meirmans, P. G. (2020). Genodive version 3.0: Easy‐to‐use software for the analysis of genetic data of diploids and polyploids. Molecular Ecology Resources, 20, 1126–1131. 32061017 10.1111/1755-0998.13145 PMC7496249

[eva13593-bib-0087] Melbourne, B. A. , & Hastings, A. (2009). Highly variable spread rates in replicated biological invasions: Fundamental limits to predictability. Science, 325, 1536–1539. 19762641 10.1126/science.1176138

[eva13593-bib-0088] Merow, C. , Smith, M. J. , & Silander, J. A., Jr. (2013). A practical guide to MaxEnt for modeling species' distributions: What it does, and why inputs and settings matter. Ecography, 36, 1058–1069.

[eva13593-bib-0089] Moody, M. E. , Mueller, L. D. , & Soltis, D. E. (1993). Genetic variation and random drift in autotetraploid populations. Genetics, 134, 649–657. 8325493 10.1093/genetics/134.2.649 PMC1205504

[eva13593-bib-0090] Moran, E. V. , Reid, A. , & Levine, J. M. (2017). Population genetics and adaptation to climate along elevation gradients in invasive Solidago canadensis. PLoS One, 12, e0185539. 28957402 10.1371/journal.pone.0185539 PMC5619793

[eva13593-bib-0091] Morente‐López, J. , Kass, J. M. , Lara‐Romero, C. , Serra‐Diaz, J. M. , Soto‐Correa, J. C. , Anderson, R. P. , & Iriondo, J. M. (2022). Linking ecological niche models and common garden experiments to predict phenotypic differentiation in stressful environments: Assessing the adaptive value of marginal populations in an alpine plant. Global Change Biology, 28, 4143–4162. 35359032 10.1111/gcb.16181 PMC9325479

[eva13593-bib-0092] Morrow, A. L. (1979). Studies on the reproductive biology of leafy spurge (Euphoriba esula). Weed Science, 27, 106–109.

[eva13593-bib-0093] Nei, M. (1972). Genetic distance between populations. The American Naturalist, 106, 283–292.

[eva13593-bib-0094] Nei, M. , Maruyama, T. , & Chakraborty, R. (1975). The bottleneck effect and genetic variability IN populations. Evolution, 29, 1–10. 28563291 10.1111/j.1558-5646.1975.tb00807.x

[eva13593-bib-0095] Oduor, A. M. O. , Leimu, R. , & van Kleunen, M. (2016). Invasive plant species are locally adapted just as frequently and at least as strongly as native plant species. Journal of Ecology, 104, 957–968.

[eva13593-bib-0096] Otto, S. P. , & Whitton, J. (2000). Polyploid incidence and evolution. Annual Review of Genetics, 34, 401–437. 10.1146/annurev.genet.34.1.401 11092833

[eva13593-bib-0098] Pandit, M. K. , Pocock, M. J. O. , & Kunin, W. E. (2011). Ploidy influences rarity and invasiveness in plants. Journal of Ecology, 99, 1108–1115.

[eva13593-bib-0099] Parmesan, C. (2006). Ecological and evolutionary responses to recent climate change. Annual Review of Ecology, Evolution, and Systematics, 37, 637–669.

[eva13593-bib-0100] Parmesan, C. , & Hanley, M. E. (2015). Plants and climate change: Complexities and surprises. Annals of Botany, 116, 849–864. 26555281 10.1093/aob/mcv169 PMC4640131

[eva13593-bib-0101] Peischl, S. , Dupanloup, I. , Kirkpatrick, M. , & Excoffier, L. (2013). On the accumulation of deleterious mutations during range expansions. Molecular Ecology, 22, 5972–5982. 24102784 10.1111/mec.12524

[eva13593-bib-0102] Pemberton, R. W. (1988). Myrmecochory in the introduced range weed, leafy spurge (Euphorbia esula L.). The American Midland Naturalist, 119, 431–435.

[eva13593-bib-0103] Peter, B. M. , & Slatkin, M. (2013). Detecting range expansions from genetic data. Evolution, 67, 3274–3289. 24152007 10.1111/evo.12202 PMC4282923

[eva13593-bib-0104] Petitpierre, B. , Broennimann, O. , Kueffer, C. , Daehler, C. , & Guisan, A. (2017). Selecting predictors to maximize the transferability of species distribution models: Lessons from cross‐continental plant invasions: Which predictors increase the transferability of SDMs? Global Ecology and Biogeography, 26, 275–287.

[eva13593-bib-0105] Petitpierre, B. , Kueffer, C. , Broennimann, O. , Randin, C. , Daehler, C. , & Guisan, A. (2012). Climatic niche shifts are rare among terrestrial plant invaders. Science, 335, 1344–1348. 22422981 10.1126/science.1215933

[eva13593-bib-0106] Phillips, S. J. , Anderson, R. P. , Dudík, M. , Schapire, R. E. , & Blair, M. E. (2017). Opening the black box: An open‐source release of Maxent. Ecography, 40, 887–893.

[eva13593-bib-0107] Phillips, S. J. , & Dudík, M. (2008). Modeling of species distributions with Maxent: New extensions and a comprehensive evaluation. Ecography, 31, 161–175.

[eva13593-bib-0108] Pina‐Martins, F. , Silva, D. N. , Fino, J. , & Paulo, O. S. (2017). Structure_threader: An improved method for automation and parallelization of programs structure, fastStructure and MavericK on multicore CPU systems. Molecular Ecology Resources, 17, e268–e274. 28776963 10.1111/1755-0998.12702

[eva13593-bib-0109] Prentis, P. J. , Wilson, J. R. U. , Dormontt, E. E. , Richardson, D. M. , & Lowe, A. J. (2008). Adaptive evolution in invasive species. Trends in Plant Science, 13, 288–294. 18467157 10.1016/j.tplants.2008.03.004

[eva13593-bib-0110] Pritchard, J. K. , Stephens, M. , & Donnelly, P. (2000). Inference of population structure using multilocus genotype data. Genetics, 155, 945–959. 10835412 10.1093/genetics/155.2.945 PMC1461096

[eva13593-bib-0153] R Development Core Team . (2015). R: A language and environment for statistical computing. R Foundation for Statistical Computing. https://www.R‐project.org/

[eva13593-bib-0111] Reed, D. H. , & Frankham, R. (2001). How closely correlated are molecular and quantitative measures of genetic variation? A meta‐analysis. Evolution, 55, 1095–1103. 11475045 10.1111/j.0014-3820.2001.tb00629.x

[eva13593-bib-0112] Rice, A. , Šmarda, P. , Novosolov, M. , Drori, M. , Glick, L. , Sabath, N. , Meiri, S. , Belmaker, J. , & Mayrose, I. (2019). The global biogeography of polyploid plants. Nature Ecology & Evolution, 3, 265–273. 30697006 10.1038/s41559-018-0787-9

[eva13593-bib-0113] Richards, C. L. , Bossdorf, O. , Muth, N. Z. , Gurevitch, J. , & Pigliucci, M. (2006). Jack of all trades, master of some? On the role of phenotypic plasticity in plant invasions. Ecology Letters, 9, 981–993. 16913942 10.1111/j.1461-0248.2006.00950.x

[eva13593-bib-0114] Riina, R. , Peirson, J. A. , Geltman, D. V. , Molero, J. , Frajman, B. , Pahlevani, A. , Barres, L. , Morawetz, J. J. , Salmaki, Y. , Zarre, S. , Kryukov, A. , Bruyns, P. V. , & Berry, P. E. (2013). A worldwide molecular phylogeny and classification of the leafy spurges, euphorbia subgenus Esula (Euphorbiaceae). Taxon, 62, 316–342.

[eva13593-bib-0115] Rochette, N. C. , Rivera‐Colón, A. G. , & Catchen, J. M. (2019). Stacks 2: Analytical methods for paired‐end sequencing improve RADseq‐based population genomics. Molecular Ecology, 28, 4737–4754. 31550391 10.1111/mec.15253

[eva13593-bib-0116] Ronfort, J. , Jenczewski, E. , Bataillon, T. , & Rousset, F. (1998). Analysis of population structure in autotetraploid species. Genetics, 150, 921–930. 9755220 10.1093/genetics/150.2.921 PMC1460367

[eva13593-bib-0117] Rozas, J. , Ferrer‐Mata, A. , Sánchez‐DelBarrio, J. C. , Guirao‐Rico, S. , Librado, P. , Ramos‐Onsins, S. E. , & Sánchez‐Gracia, A. (2017). DnaSP 6: DNA Sequence Polymorphism Analysis of Large Data Sets. Mol Biol Evol., 34(12), 3299–3302. 29029172 10.1093/molbev/msx248

[eva13593-bib-0118] Rutland, C. A. , Hall, N. D. , & McElroy, J. S. (2021). The impact of polyploidization on the evolution of weed species: Historical understanding and current limitations. Frontiers in Agronomy, 3, 626454.

[eva13593-bib-0119] Sakai, A. K. , Allendorf, F. W. , Holt, J. S. , Lodge, D. M. , Molofsky, J. , With, K. A. , Baughman, S. , Cabin, R. J. , Cohen, J. E. , Ellstrand, N. C. , McCauley, D. E. , O'Neil, P. , Parker, I. M. , Thompson, J. N. , & Weller, S. G. (2001). The population biology of invasive specie. Annual Review of Ecology and Systematics, 32, 305–332.

[eva13593-bib-0120] Schulz‐Schaeffer, J. , & Gerhardt, S. (1989). Cytotaxonomic analysis of the euphorbia spp leafy spurge complex ii. comparative study of the chromosome morphology. Biol Zent Bl, 108, 69–76.

[eva13593-bib-0121] Searcy, C. A. , & Shaffer, H. B. (2016). Do ecological niche models accurately identify climatic determinants of species ranges? The American Naturalist, 187, 423–435. 10.1086/685387 27028071

[eva13593-bib-0122] Selechnik, D. , Richardson, M. F. , Shine, R. , DeVore, J. L. , Ducatez, S. , & Rollins, L. A. (2019). Increased adaptive variation despite reduced overall genetic diversity in a rapidly adapting invader. Frontiers in Genetics, 10, 1221. 31850072 10.3389/fgene.2019.01221 PMC6901984

[eva13593-bib-0123] Selleck, G. W. , Coupland, R. T. , & Frankton, C. (1962). Leafy spurge in Saskatchewan. Ecological Monographs, 32, 1–29.

[eva13593-bib-0124] Sexton, J. P. , McKay, J. K. , & Sala, A. (2002). Plasticity and genetic diversity may allow Saltcedar to invade cold climates in North America. Ecological Applications, 12, 1652–1660.

[eva13593-bib-0125] Singmann, H. , Bolker, B. , Westfall, J. , & Aust, F. (2016). afex: Analysis of factorial experiments. Version 0.16‐1 https://CRAN.R‐project.org/package=afex

[eva13593-bib-0126] Slatkin, M. (1987). Gene flow and the geographic structure of natural populations. Science, 236, 787–792. 3576198 10.1126/science.3576198

[eva13593-bib-0127] Slatkin, M. (1993). Isolation by distance in equilibrium and non‐equilibrium populations. Evolution, 47, 264–279. 28568097 10.1111/j.1558-5646.1993.tb01215.x

[eva13593-bib-0128] Slatkin, M. , & Excoffier, L. (2012). Serial founder effects during range expansion: A spatial analog of genetic drift. Genetics, 191, 171–181. 22367031 10.1534/genetics.112.139022 PMC3338258

[eva13593-bib-0129] Soltis, P. S. , & Soltis, D. E. (2000). The role of genetic and genomic attributes in the success of polyploids. Proceedings of the National Academy of Sciences of the United States of America, 97, 7051–7057. 10860970 10.1073/pnas.97.13.7051 PMC34383

[eva13593-bib-0130] Suarez, A. V. , & Tsutsui, N. D. (2008). The evolutionary consequences of biological invasions. Molecular Ecology, 17, 351–360. 18173507 10.1111/j.1365-294X.2007.03456.x

[eva13593-bib-0131] Sullivan, L. L. , Li, B. , Miller, T. E. X. , Neubert, M. G. , & Shaw, A. K. (2017). Density dependence in demography and dispersal generates fluctuating invasion speeds. Proceedings of the National Academy of Sciences of the United States of America, 114, 5053–5058. 28442569 10.1073/pnas.1618744114 PMC5441710

[eva13593-bib-0132] Tabassum, S. , & Leishman, M. R. (2018). Have your cake and eat it too: Greater dispersal ability and faster germination towards range edges of an invasive plant species in eastern Australia. Biological Invasions, 20, 1199–1210.

[eva13593-bib-0133] Tajima, F. (1989). Statistical method for testing the neutral mutation hypothesis by DNA polymorphism. Genetics, 123, 585–595. 2513255 10.1093/genetics/123.3.585 PMC1203831

[eva13593-bib-0134] te Beest, M. , Le Roux, J. J. , Richardson, D. M. , Brysting, A. K. , Suda, J. , Kubesová, M. , & Pysek, P. (2012). The more the better? The role of polyploidy in facilitating plant invasions. Ann Bot., 109(1), 19–45. 22040744 10.1093/aob/mcr277 PMC3241594

[eva13593-bib-0135] Travis, J. M. J. , Mynard, P. , & Bocedi, G. (2021). Evolution of reduced dormancy during range expansions. *bioRxiv* 2021.10.11.463894.

[eva13593-bib-0136] Treier, U. A. , Broennimann, O. , Normand, S. , Guisan, A. , Schaffner, U. , Steinger, T. , & Müller‐Schärer, H. (2009). Shift in cytotype frequency and niche space in the invasive plant Centaurea maculosa. Ecology, 90, 1366–1377. 19537556 10.1890/08-0420.1

[eva13593-bib-0137] Uller, T. , & Leimu, R. (2011). Founder events predict changes in genetic diversity during human‐mediated range expansions. Global Change Biology, 17, 3478–3485.

[eva13593-bib-0138] van Boheemen, L. A. , Atwater, D. Z. , & Hodgins, K. A. (2019). Rapid and repeated local adaptation to climate in an invasive plant. The New Phytologist, 222, 614–627. 30367474 10.1111/nph.15564

[eva13593-bib-0139] Van de Peer, Y. , Ashman, T.‐L. , Soltis, P. S. , & Soltis, D. E. (2021). Polyploidy: An evolutionary and ecological force in stressful times. Plant Cell, 33, 11–26. 33751096 10.1093/plcell/koaa015 PMC8136868

[eva13593-bib-0140] Verma, P. , & Majee, M. (2013). Seed germination and viability test in tetrazolium (TZ) assay. Bio‐Protocol, 3, 1–4.

[eva13593-bib-0141] Wang, D. , Xu, X. , Zhang, H. , Xi, Z. , Abbott, R. J. , Fu, J. , & Liu, J. (2022). Abiotic niche divergence of hybrid species from their progenitors. The American Naturalist, 200, 634–645. 10.1086/721372 36260852

[eva13593-bib-0142] Warren, D. L. , Glor, R. E. , & Turelli, M. (2008). Environmental niche equivalency versus conservatism: Quantitative approaches to niche evolution. Evolution, 62, 2868–2883. 18752605 10.1111/j.1558-5646.2008.00482.x

[eva13593-bib-0143] Welles, S. R. , & Dlugosch, K. M. (2019). Population genomics of colonization and invasion. In O. P. Rajora (Ed.), Population genomics: Concepts, approaches and applications (pp. 655–683). Springer International Publishing.

[eva13593-bib-0144] West, N. M. , Gaskin, J. F. , Milan, J. , & Rand, T. A. (2023). High genetic diversity in the landscape suggests frequent seedling recruitment by Euphorbia virgata Waldst. & Kit. (leafy spurge) in the northern U.S.A. Biological Invasions, 25, 645–652.

[eva13593-bib-0145] Westerband, A. C. , Funk, J. L. , & Barton, K. E. (2021). Intraspecific trait variation in plants: A renewed focus on its role in ecological processes. Annals of Botany, 127, 397–410. 33507251 10.1093/aob/mcab011 PMC7988520

[eva13593-bib-0146] Wicks, G. A. , & Derscheid, L. A. (1958). Leafy spurge seed maturation studies. Proceedings of North Central Weed Control Conference.

[eva13593-bib-0147] Williams, J. L. , Kendall, B. E. , & Levine, J. M. (2016). Rapid evolution accelerates plant population spread in fragmented experimental landscapes. Science, 353, 482–485. 27471303 10.1126/science.aaf6268

[eva13593-bib-0148] Wolkovich, E. M. , Davies, T. J. , Schaefer, H. , Cleland, E. E. , Cook, B. I. , Travers, S. E. , Willis, C. G. , & Davis, C. C. (2013). Temperature‐dependent shifts in phenology contribute to the success of exotic species with climate change. American Journal of Botany, 100, 1407–1421. 23797366 10.3732/ajb.1200478

[eva13593-bib-0149] Woods, E. C. , & Sultan, S. E. (2022). Post‐introduction evolution of a rapid life‐history strategy in a newly invasive plant. Ecology, 103, e3803. 35796712 10.1002/ecy.3803

[eva13593-bib-0150] Wright, S. (1943). Isolation by distance. Genetics, 28, 114–138. 17247074 10.1093/genetics/28.2.114 PMC1209196

